# Structure of Human Enterovirus 70 and Its Inhibition by Capsid-Binding Compounds

**DOI:** 10.1128/jvi.00604-22

**Published:** 2022-08-08

**Authors:** Tibor Füzik, Jana Moravcová, Sergei Kalynych, Pavel Plevka

**Affiliations:** a Structural Virology, Central European Institute of Technology, Masaryk University, Brno, Czech Republic; University of Kentucky College of Medicine

**Keywords:** virus, acute hemorrhagic conjunctivitis, human, enterovirus, *Picornavirales*, *Picornaviridae*, virion, structure, capsid, protein, jelly roll, inhibitor, antiviral, canyon, virion structure

## Abstract

Enterovirus 70 (EV70) is a human pathogen belonging to the family *Picornaviridae*. EV70 is transmitted by eye secretions and causes acute hemorrhagic conjunctivitis, a serious eye disease. Despite the severity of the disease caused by EV70, its structure is unknown. Here, we present the structures of the EV70 virion, altered particle, and empty capsid determined by cryo-electron microscopy. The capsid of EV70 is composed of the subunits VP1, VP2, VP3, and VP4. The partially collapsed hydrophobic pocket located in VP1 of the EV70 virion is not occupied by a pocket factor, which is commonly present in other enteroviruses. Nevertheless, we show that the pocket can be targeted by the antiviral compounds WIN51711 and pleconaril, which block virus infection. The inhibitors prevent genome release by stabilizing EV70 particles. Knowledge of the structures of complexes of EV70 with inhibitors will enable the development of capsid-binding therapeutics against this virus.

**IMPORTANCE** Globally distributed enterovirus 70 (EV70) causes local outbreaks of acute hemorrhagic conjunctivitis. The discharge from infected eyes enables the high-efficiency transmission of EV70 in overcrowded areas with low hygienic standards. Currently, only symptomatic treatments are available. We determined the structures of EV70 in its native form, the genome release intermediate, and the empty capsid resulting from genome release. Furthermore, we elucidated the structures of EV70 in complex with two inhibitors that block virus infection, and we describe the mechanism of their binding to the virus capsid. These results enable the development of therapeutics against EV70.

## INTRODUCTION

Enteroviruses are vertebrate and human pathogens that cause diseases ranging from mild respiratory illnesses to life-threatening meningitis (1). Human enterovirus 70 (EV70), unlike other enteroviruses, replicates in conjunctival and corneal epithelium cells of the eye and leads to acute hemorrhagic conjunctivitis. The most common symptoms are swelling, congestion, watering, and pain in the eyes as well as subconjunctival and eye globe hemorrhage (2). The first identified outbreak of EV70 dates back to 1969 in West Africa (3). EV70, which is highly contagious, is transmitted by eye secretions and regularly causes large-scale outbreaks in tropical countries; however, local outbreaks have been reported all over the world (4–9). In exceptional cases, patients develop secondary neurological symptoms resembling those of polio-like syndrome (10–12). Although some parts of the EV70 VP1 protein were found to be immunogenic (13, 14), no effective vaccination against EV70 has been developed. Furthermore, no antiviral drugs inhibiting infection by EV70 are available.

EV70 belongs to the species *Enterovirus D* of the genus *Enterovirus* of the family *Picornaviridae*. The genome of EV70 is formed by positive-sense, single-stranded RNA that is 7,400 nucleotides long (15). Capsids of enteroviruses are built from 60 copies of the virus proteins VP1 to -3 that form an icosahedral shell with pseudo-T=3 symmetry. The capsid proteins VP1, VP2, and VP3 have the jelly roll β-sandwich fold shared by picornaviruses and numerous viruses from other families (16). The eight β-strands that constitute the core of the β-sandwich are conventionally named B to I, and the two antiparallel β-sheets contain strands BIDG and CHEF (16). Sixty copies of the minor capsid protein VP4 cover the inner surface of the capsid (16, 17). Deep clefts called “canyons” encircle the 5-fold icosahedral axes of enterovirus capsids (16).

Receptors utilized by enteroviruses can be classified as (i) attachment receptors that enable the binding of virus particles to a cell surface and (ii) uncoating receptors that, in addition to cell binding, induce the release of virus genomes (18). EV70 uses decay-accelerating factor (DAF)/CD55 as an attachment receptor to infect HeLa cells (19, 20). It was shown previously for other enteroviruses that DAF does not bind to the canyon and does not induce genome release (21–24). Some human leukocyte cell lines were shown to be permissive to EV70 even though they do not express DAF, indicating that the virus can utilize another receptor (25). It was shown that the glycosylation of cell surface proteins and specifically sialic acid is required for EV70 cell attachment (26, 27). The uncoating receptors commonly bind to the canyons of enterovirus capsids (28, 29). Among enteroviruses, these include intracellular Adhesion Molecule 1 (ICAM1) (30), neonatal Fc receptor (31), scavenger receptor class B member 2 (SCARB2) (32), coxsackievirus-adenovirus receptor (CAR) (33), and CD155 (34). However, none of these receptors were shown to bind EV70.

The VP1 subunits of most enteroviruses contain cavities that are filled with hydrophobic molecules called pocket factors (35–40). The binding of enteroviruses to uncoating receptors with an immunoglobulin fold induces the expulsion of the pocket factors from the hydrophobic pockets (35, 38, 41). Receptor binding and exposure to acidic pH in the endosome induce the conversion of enterovirus virions to altered particles, which are characterized by collapsed hydrophobic pockets in VP1, the absence of pocket factors, expanded capsids, the release of VP4 subunits, the externalization of the N termini of VP1 subunits, and genome reorganization, relative to native virions (42, 43). The altered particles spontaneously release their genomes either through pores positioned at 2-fold symmetry axes of their capsid (42, 44–48) or by capsid opening (43).

Enterovirus genome release can be inhibited by small-molecule pocket-binding inhibitors (49). Numerous “WIN compounds” were developed by Sterling-Winthrop, of which WIN51711 (also known as disoxaril) was broadly effective against enteroviruses (50–52). Modifications of WIN51711 to improve its stability, water solubility, and antienteroviral activity led to the creation of pleconaril (WIN63843) (53). It has been shown that these pocket-binding compounds prevent the activation of particles and genome release (40, 49, 54, 55).

Here, we present the structures of the virion, altered particle, and empty capsid of EV70. In addition, we also determined the structures of EV70 in complex with the capsid-binding inhibitors WIN51711 and pleconaril and show that these compounds overstabilize the virus particles and reduce their infectivity. Knowledge of virus–inhibitor interactions enables the design and development of new anti-EV70 drugs.

## RESULTS AND DISCUSSION

### Structure of the EV70 virion and capsid proteins.

**FIG 1:**
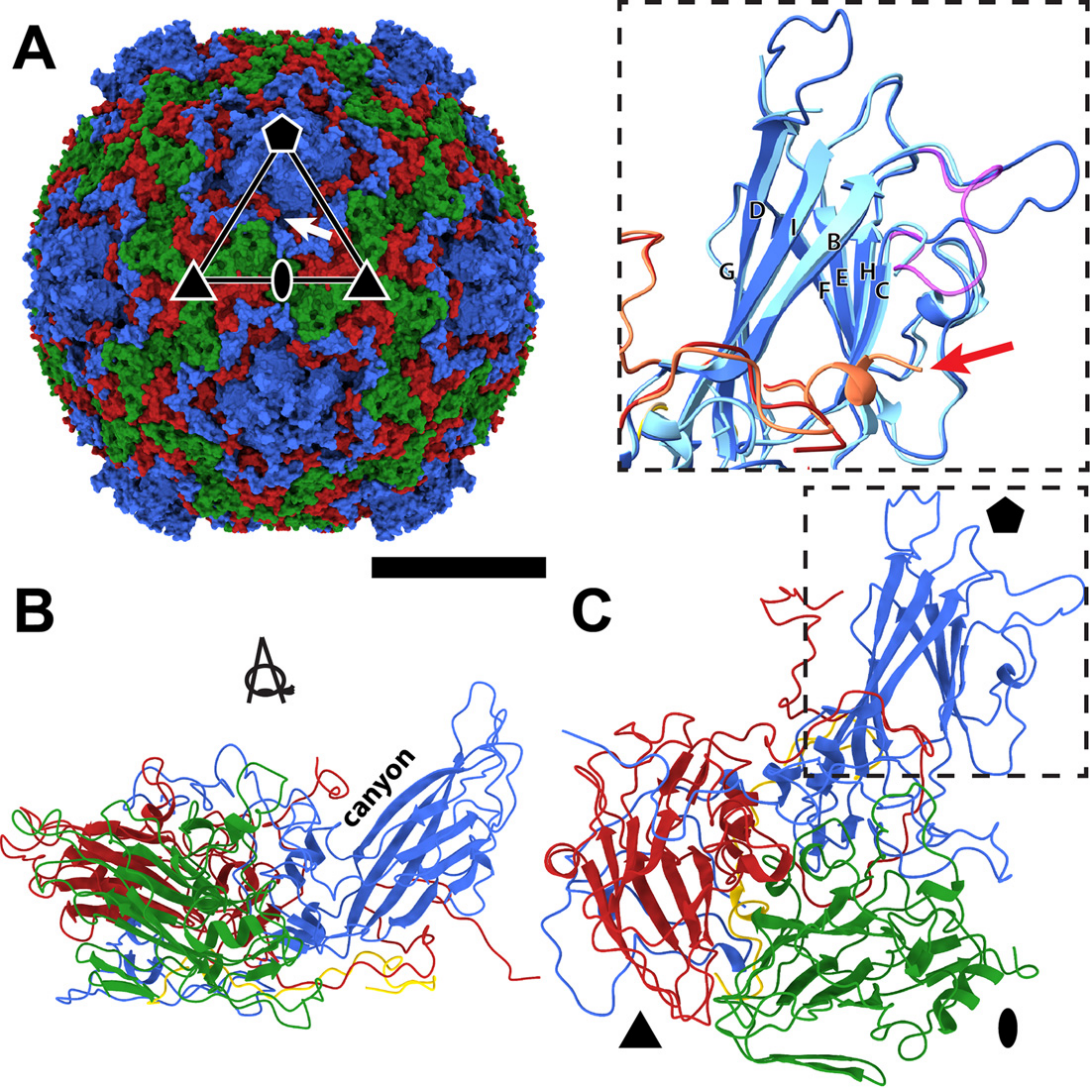
Virion structure of EV70. (A) Molecular surface of the EV70 virion colored according to capsid proteins. VP1 subunits are shown in blue, VP2 subunits are in green, and VP3 subunits are in red. The positions of selected icosahedral symmetry axes are indicated with an oval for 2-fold, a triangle for 3-fold, and a pentagon for 5-fold. An asymmetric unit is outlined with a triangle. The white arrow indicates the position of an opening into a selected VP1 pocket. Bar, 10 nm. (B and C) Cartoon representations of the protomer of capsid proteins in two orientations related by 90° rotation along the *x* axis and 90° rotation along the *y* axis. The coloring of the subunits is the same as that described above for panel A, and the VP4 subunit is shown in yellow. The inset of panel C shows the differences between the BC and DE loops of VP1 and the C termini of VP3 (red arrow) of EV70 (dark colors), EV-D68 strain Fermon (light colors), and EV-D68 strain MO (magenta).

**FIG 2:**
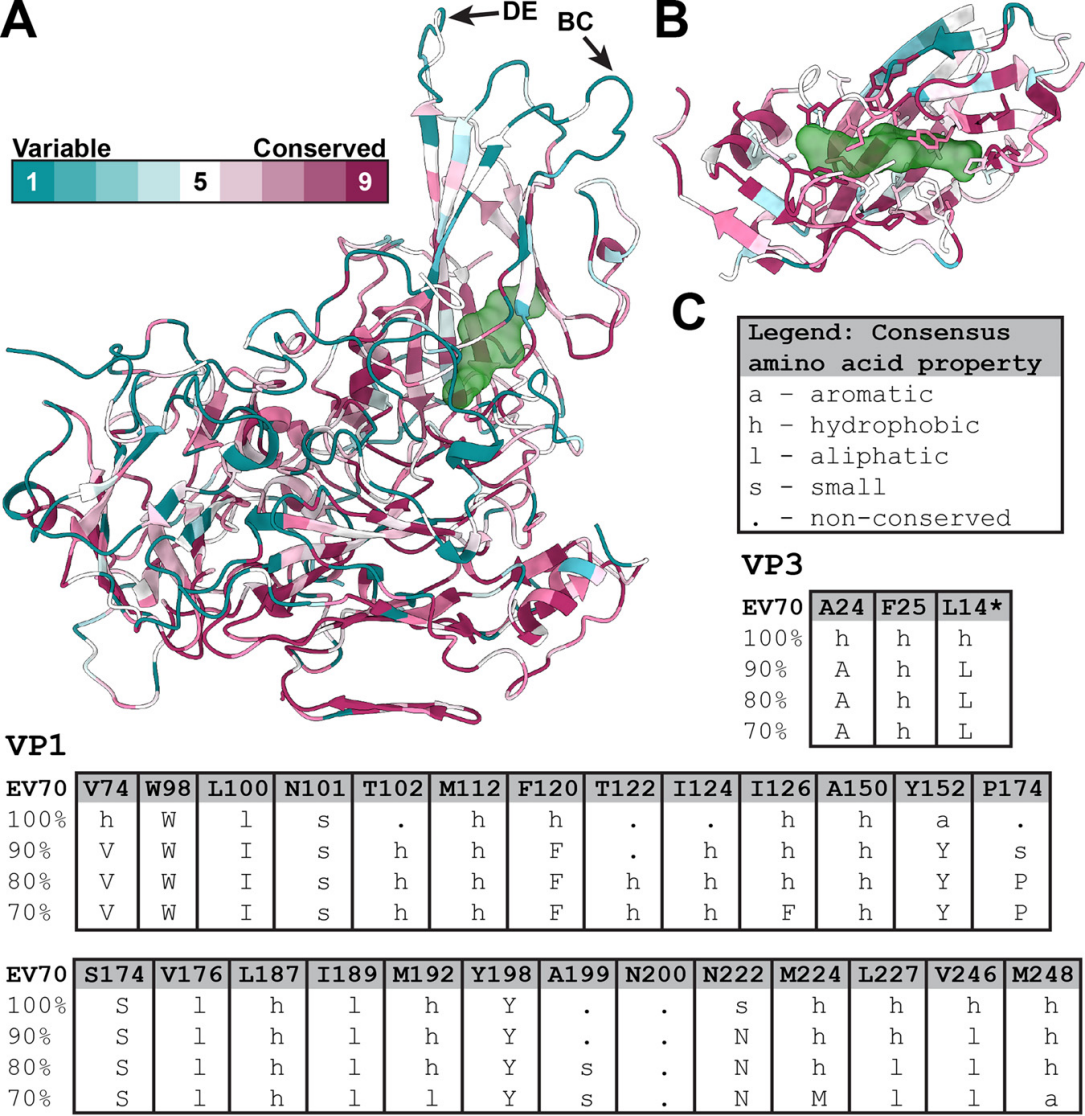
Conservation of pocket-forming residues among enteroviruses. (A) Cartoon representation of the EV70 protomer colored according to sequence conservation among 30 selected enteroviruses (for details, see Fig. S8 in the supplemental material) (85). The position of the VP1 pocket with pleconaril is highlighted, represented as a green semitransparent molecular surface. The arrows indicate the BC and DE loops of VP1, which are part of antigenic sites in enteroviruses. (B) Detail of the VP1 pocket with side chains of residues shown in a stick representation. (C) Conservation of pocket-forming residues of VP1 and VP3 (the asterisk denotes a residue contributing to a pocket in a neighboring protomer). The table shows the conservation of amino acid residues among 30 selected enteroviruses. The consensus of residues at the levels of 70% to 100% is indicated. Capital letters indicate consensus amino acid residues, and lowercase letters indicate classes of residues with specific physicochemical properties (86).

Cryo-electron microscopy (cryo-EM) was used to determine a three-dimensional (3D) reconstruction of the virion of EV70 to a resolution of 2.63 Å (Fig. 1; see also Table S1 and Fig. S1 in the supplemental material). The nonenveloped capsid of EV70 has a diameter of 30 nm. The map enabled the building of the structure of capsid proteins except for residues 1 to 6 and 304 to 306 of VP1, 1 to 10 and 249 to 250 of VP2, and 1 to 27 and 60 to 68 of VP4. The major capsid proteins of EV70 are organized with pseudo-T=3 icosahedral symmetry, with VP1 subunits forming pentamers around 5-fold symmetry axes and VP2 and VP3 subunits forming heterohexamers at 3-fold axes of the capsid (Fig. 1A). EV70 has the highest sequence identity to EV-D68 (76%) of the viruses that have been structurally characterized to date (35). In contrast to EV-D68, the structure of which was solved using X-ray crystallography (35) and cryo-EM (56), our reconstruction of EV70 enabled the building of the DE loop of VP1 that forms a protrusion around the 5-fold symmetry axes of the capsid (Fig. 1C). The DE and BC loops of VP1 are highly variable in their amino acid sequences among enteroviruses (Fig. 2A and Fig. S2), and it was shown previously that they are part of antigenic sites (57–62). The lower local resolution of the cryo-EM map and higher-temperature factors of the atoms from the amino acids forming the BC and DE loops of VP1 indicate that they are flexible (Fig. S3 and S4). Furthermore, the VP3 subunit of EV-D68 forms a 7-residue-long C-terminal α-helix, which is not present in EV70 (Fig. 1C). Because of the absence of the helix, the canyon of EV70 is deeper than that of EV-D68 (Fig. S5).

**FIG 3:**
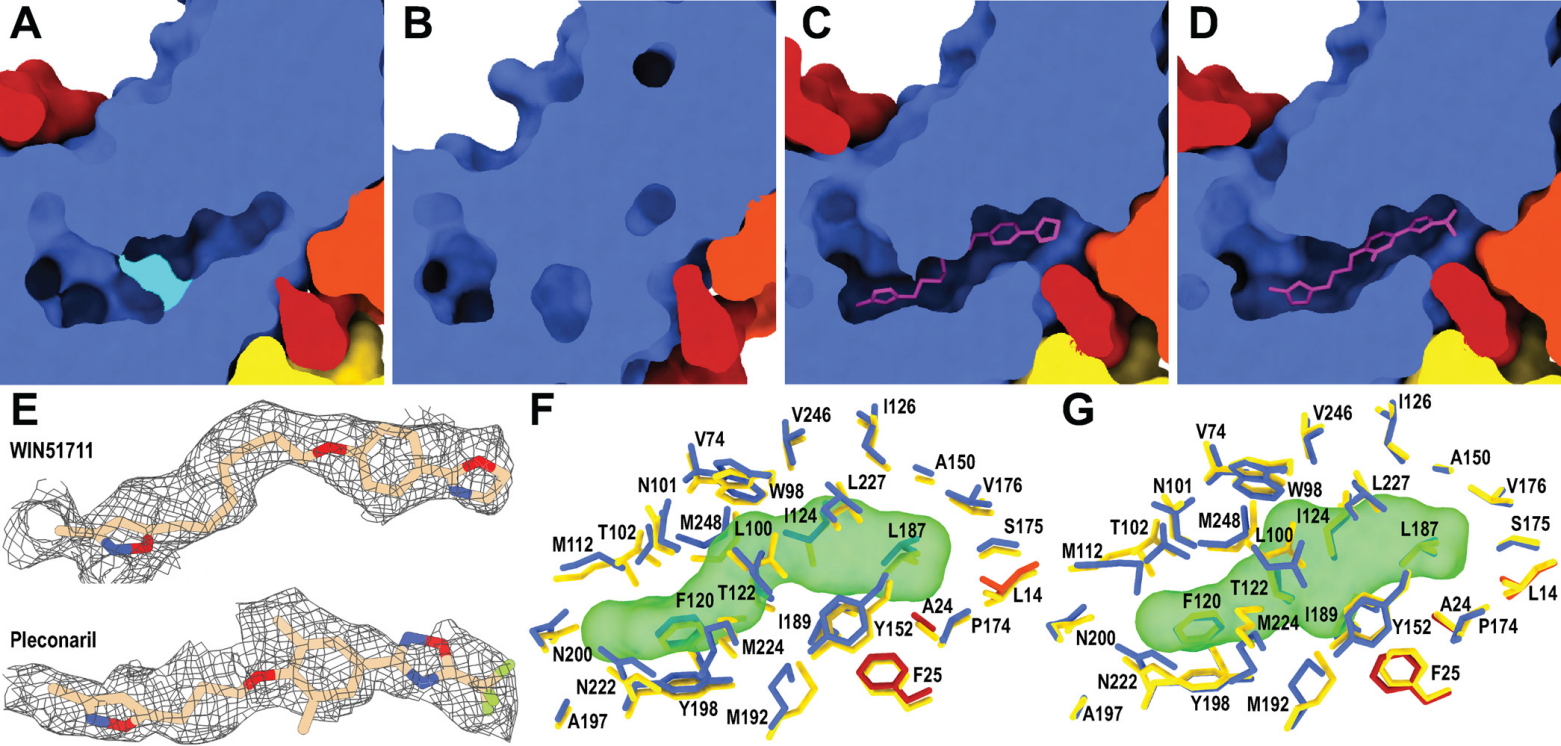
The pocket in VP1 of EV70 and its interactions with capsid-binding inhibitors. (A to D) Molecular surface representations of capsid proteins clipped to show the VP1 pocket of EV70. (A and B) The pocket is partially blocked by Met224 of VP1 (in cyan) in the virion (A) and completely collapsed in the altered particle (B). (C and D) The binding of WIN51711 (C) and pleconaril (D) induces the expansion of the pocket. The inhibitors are shown in a stick representation. The VP1 subunit is shown in blue, VP3 is in red, VP3 from a neighboring protomer is in orange, and inhibitors are in magenta. (E) Cryo-EM maps showing inhibitors bound in the VP1 pocket of EV70. The inhibitors are shown in a stick representation. (F and G) Stick representations of the interactions of the side chains of residues with WIN51711 (F) and pleconaril (G). The inhibitors are shown as semitransparent molecular surfaces. The inhibitors interact with the same residues.

The VP1 subunits of most enteroviruses contain hydrophobic pockets positioned between the β-sheets BIDG and CHEF, which form the core of the subunit (Fig. 1B and C) (16). The opening of the pocket is located approximately between 5-fold and 2-fold axes of icosahedral symmetry (Fig. 1A). The pockets of most enteroviruses are occupied by hydrophobic pocket factors, most likely fatty acids or lipids (35–40). In contrast, the pocket in VP1 of EV70 does not contain a continuous density corresponding to the pocket factor. It cannot be excluded that the pocket factor was lost from EV70 virions during the purification process; however, other enteroviruses that contain pocket factors were purified using the same or a very similar procedure (35, 40, 43). The protocols differed only in the strains of the cell lines used for virus production. VP1 pockets in native virions of human rhinovirus serotype 14 (HRV14) and HRV3 are empty and collapsed (54, 63). In contrast, the pocket in EV70 VP1 is not completely collapsed, but the side chain of Met224 of VP1 partially blocks its central part (Fig. 3A and Fig. S6). Thus, the discontinuous density fragments inside the pocket may correspond to solvent molecules. Met221, which is homologous to Met224 of VP1 of EV70, blocks the entrance of the hydrophobic pockets of HRV3 and HRV14, preventing the binding of a lipidic pocket factor (54, 63). The structure of EV-D68 strain Fermon, solved by X-ray crystallography, contained a pocket factor (35), whereas the structure of EV-D68 strain MO (US/MO/14-18947), solved by cryo-EM, lacked it (56). The empty pocket of EV-D68 strain MO was partially collapsed by the movement of the GH loop of VP1 pushing Ile217 into the pocket cavity. In EV70, the side chain of Met224 occupies the same space as that of the dislocated Ile217 in EV-D68 (Fig. S7); however, we did not observe any movement of the VP1 GH loop analogous to that in EV-D68 (Fig. S7). Whereas the main part of the EV70 pocket is formed by 26 residues of the VP1 subunit, the end of the pocket pointing toward the virion center is capped by residues from two VP3 subunits (Fig. 3A). One of the VP3 subunits belongs to the same protomer as the VP1 subunit, and the second VP3 subunit belongs to a neighboring protomer (Fig. 3A, F, and G). Therefore, the pocket is accessible only from the outside of the virus particle. A comparison of the amino acid sequences of the capsid proteins of 30 enteroviruses demonstrated that the residues forming the pockets are more conserved than the surface residues (Fig. 2 and Fig. S2 and S8) and are mostly hydrophobic or aliphatic (Fig. 2C and Fig. S8). Completely conserved residues that form the pocket include Trp98, Ser174, and Tyr198 of VP1 (EV70 numbering).

### Structural changes of EV70 capsid associated with genome release.

**FIG 4:**
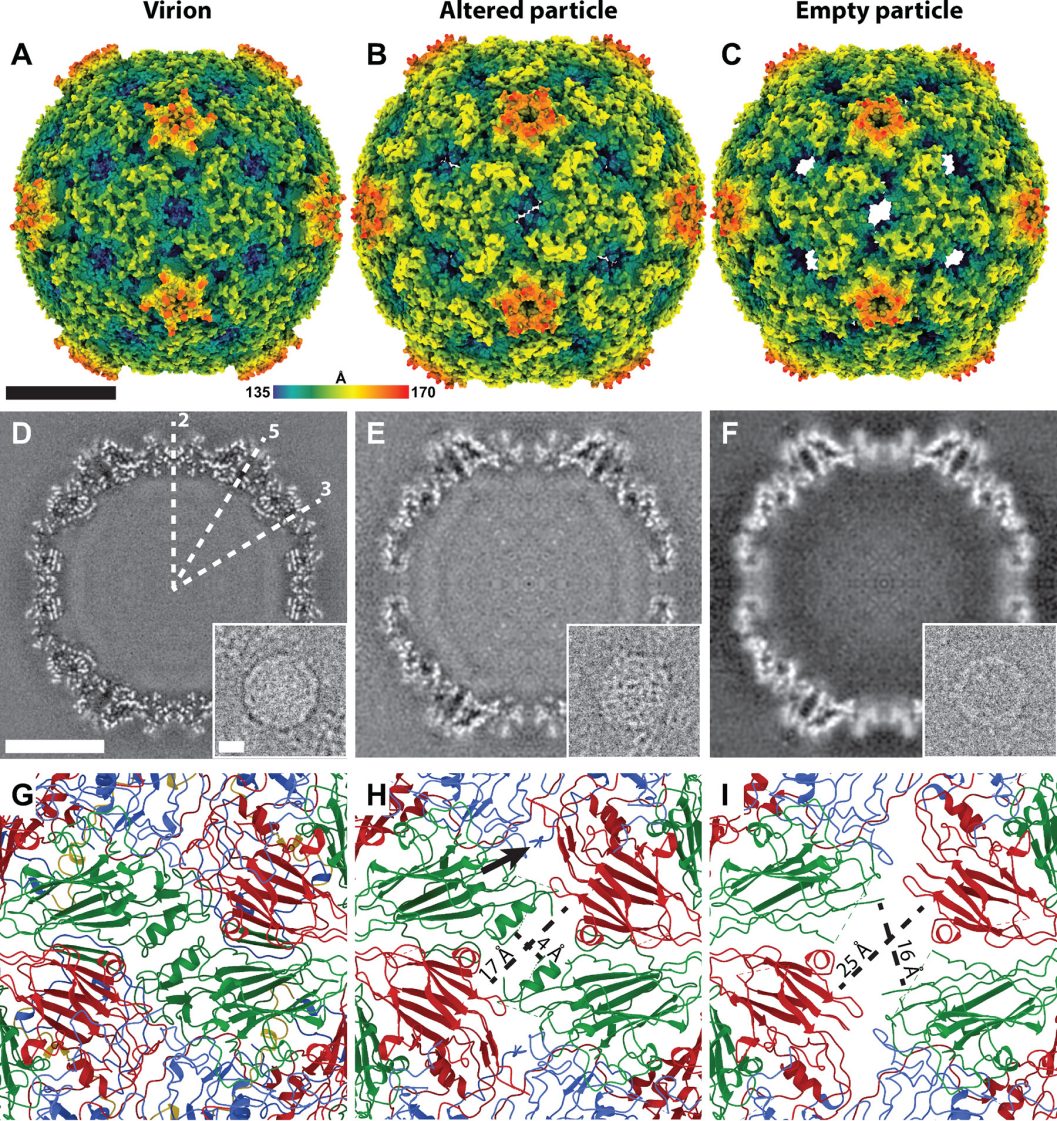
Structural comparison of the virion, altered particle, and empty particle of EV70. (A to C) Molecular surfaces rainbow-colored according to the distance from the particle center of the virion (A), altered particle (B), and empty particle (C). Altered and empty particles contain pores on the 2-fold axes of their symmetry. (D to F) Central slices of cryo-EM maps of the virion (D), altered particle (E), and empty particle (F). The insets show electron micrographs of the corresponding particles. The positions of selected icosahedral symmetry axes are indicated in panel D. (G to I) Cartoon representations of capsid proteins around the 2-fold symmetry axes of the virion (G), altered particle (H), and empty particle (I). The formation of a pore around the 2-fold symmetry axis in altered (H) and empty (I) particles is enabled by the reduction in interpentamer contacts and the loss of structure of parts of VP2 subunits. The sizes of the pores are shown in panels H and I. The arrow in panel H indicates the externalization of the N-terminal part of VP1 through a pore above the 2-fold symmetry axis in altered particles. VP1 subunits are shown in blue, VP2 subunits in green, and VP3 subunits in red. Bars indicated 10nm.

Electron micrographs of EV70 contained images of virions in their native conformation but also those of altered and empty particles (Fig. 4). Eighty-eight percent of EV70 particles contained the genome under physiological pH, whereas 12% were empty. EV70 is an acid-stable enterovirus (64, 65), although an acid-labile strain has also been reported (65). The untimely activation of EV70 occurring outside a target cell is detrimental to the virus since it decreases the number of infectious particles. For acid-labile EV-D68 (strain MO), it was shown that the virus preparation at neutral pH contained >95% genome-containing particles (56), indicating that EV-D68 is more stable at neutral pH than EV70. The most apparent difference between EV70 virions and altered particles in electron micrographs is in the distribution of their genomes (Fig. 4D and E). Whereas the genomic RNAs uniformly fill the capsids of virions, the genomes in altered particles exhibit regions of high and low density. Similar changes in the genome structure associated with particle activation have been reported for HRV2 and echovirus 18 (E18) (43, 66).

The structures of altered and empty particles of EV70 were determined to resolutions of 3.7 and 4.3 Å, respectively (Table S1). The altered particle lacks VP4 subunits and is expanded 4% in diameter compared to the native virion (Fig. 4A and B and Fig. S9). The expansion is enabled by the loosening of contacts between the pentamers of capsid protein protomers, which is accompanied by a reduction in the interpentamer binding interface from 3,350 to 2,750 Å^2^. The first 48 N-terminal residues and loops BC (residues 84 to 91) and DE (residues 134 to 142) of VP1, loop AB (residues 43 to 57) of VP2, and loops BC (residues 73 to 78) and GH (residues 170 to 172) of VP3 are not resolved in the cryo-EM reconstruction of the altered particle (Fig. S10). Amino acid residues 49 to 56 from the N terminus of VP1 cross the capsid of the altered particle of EV70, resulting in the exposure of the N terminus of VP1 at the surface of the altered particle (Fig. 4H and Fig. S11). The N termini of the VP1 subunits pass through the capsid approximately between a 2-fold axis of the altered particle and the canyon (Fig. 4H and Fig. S11). The externalization of the N terminus of VP1 is enabled by a conformational change and a shift of the GH loop of VP3 (Fig. S10 and S11) away from an interprotomer interaction interface (Fig. S11). The externalization of the N-terminal part of the VP1 subunits was observed previously in altered particles of a number of enteroviruses (31, 43, 67, 68). There is evidence that the externalization of the N termini of VP1 subunits enables the binding of enteroviruses to biological membranes (42, 69). Antibodies produced against the N-terminal part of EV70 VP1 were able to neutralize EV70 virions (13), and polyclonal anti-EV70 mouse serum was highly reactive against the N-terminal region of VP1 (14). This indicates that the externalization of the N-terminal part of EV70 VP1 occurs *in vivo*.

The pocket inside the VP1 subunit in the altered particle of EV70 is collapsed (Fig. 3B), as is also the case in altered particles of other enteroviruses that were characterized previously (42, 67). The collapse of the pocket of EV70 is enabled by the movement of VP1 strands B, C, H, and I into the pocket (Fig. S10). The altered particle of EV70 contains approximately rectangular pores of 17 by 4 Å positioned on 2-fold symmetry axes of the capsid (Fig. 4H). It was hypothesized that homologous pores serve as sites of exit for VP4 from the particles of enteroviruses (42).

The empty particle of EV70 is structurally similar to the altered particle but has the interpentamer binding interface decreased to 1,650 Å^2^ and the rectangular pores on 2-fold axes of the empty particle expanded to 25 by 16 Å (Fig. 4C, F, and I). This pore expansion is caused mostly by the loss of the structure of a helix formed by residues 90 to 98 of VP2 (Fig. 4I). Nevertheless, the residues forming the helix, even though flexible, are still located in the vicinity of the pore. The best-resolved part of the capsid of the empty particle is the intrapentamer interface formed by VP1 subunits, showing that the contacts within a pentamer are more stable than those between pentamers (Fig. S4). The mechanism of genome release of EV70 remains to be determined. Although the pore located on a 2-fold axis of the empty particle may enable the exit of a single-stranded RNA, as was proposed previously for EV-D68 (56), we cannot rule out that genome release is enabled by particle opening, as was proposed previously for echoviruses 18 and 30 (43). Because of the low number of images of empty EV70 particles in our data set, we were unable to identify the presence of open particles in the sample.

### Inhibition of EV70 infection by WIN51711 and pleconaril.

**FIG 5:**
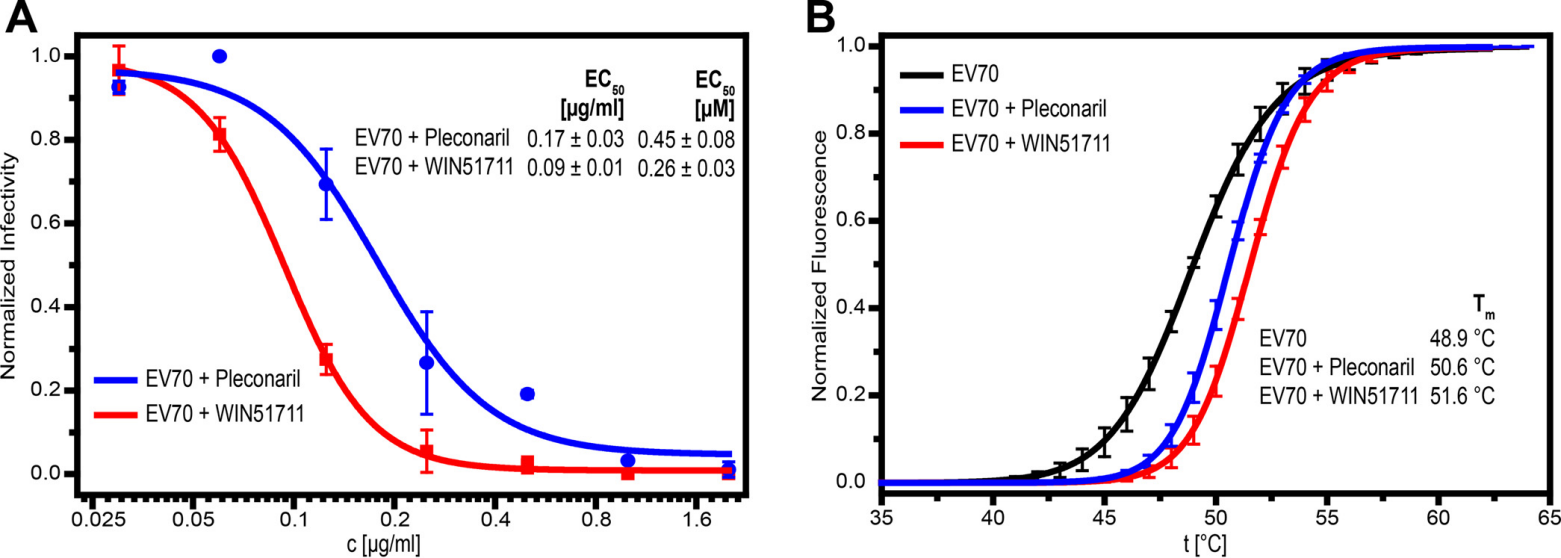
Effects of capsid-binding inhibitors on the infectivity and particle stability of EV70. (A) The reduction in the infectivity of EV70 was assayed on rPE1 cells using a plaque assay. Sigmoidal dose-response curves were fitted to the experimental data and EC_50_ values of the inhibitors were estimated. (B) Denaturation curves from thermal stability assays performed on native EV70 and EV70 mixed with capsid-binding inhibitors (15-fold molar excess of the inhibitor per binding site). As the temperature increased, the structure of the particle relaxed, and the SYBR green present in the solution interacted with genomic RNA, which caused the increase in fluorescence. EV70 particles in the presence of capsid-binding inhibitors exhibit higher capsid stability and, thus, a higher particle denaturation temperature (*T_m_*).

Capsid-binding compounds were shown previously to be potent inhibitors of enteroviruses, including those that, like EV70, do not contain pocket factors (40, 51, 53). Here, we show that WIN51711 and pleconaril (Fig. S12) inhibit EV70 with 50% effective concentration (EC_50_) values of 0.26 μM and 0.45 μM, respectively (Fig. 5A). These concentrations are within the range of previously reported EC_50_ values of other enteroviruses (Table S2) and very close to those previously reported for EV70–WIN51711 and EV-D68–pleconaril. We found that the binding of the inhibitors increases the melting temperature (*T_m_*) of EV70 from 48.9°C for the virion to 51.6°C and 50.6°C for particles treated with WIN51711 and pleconaril, respectively (Fig. 5B). The binding of WIN51711 stabilizes the particles of EV70 more than that of pleconaril, which is in agreement with its stronger inhibitory effect. Overstabilization of the particles, which prevents genome release, was proposed to be the inhibition mechanism of capsid-binding inhibitors of enteroviruses (35, 40, 70).

Because the virions of EV70 in their native conformation do not contain pocket factors, the binding of inhibitory compounds requires conformational changes to the VP1 pocket. Cryo-EM reconstructions of complexes of EV70 with WIN51711 and pleconaril were determined to resolutions of 2.74 Å and 2.31 Å, respectively (Fig. S1). The quality of the cryo-EM maps enabled the unambiguous fitting of the lateral orientation of the inhibitors into the corresponding densities (Fig. 3E to G). The binding of the inhibitors induced the expansion of the VP1 pocket, which required a 1-Å movement of residues Met112, Met192, and Met224 of VP1 away from the core of the subunit relative to the structure of the subunit in the native virion (Fig. S6b and c). Notably, the side chain of Met224, which partially blocked the pocket in the native particle, now adopts a conformation that allows the presence of the inhibitor without changing the position of the VP1 GH loop backbone, in contrast to EV-D68, where the presence of the inhibitor induces changes in the backbone (Fig. S7b). Changes in the structures of the pockets induced by the binding of inhibitors were reported previously for EV-D68, HRV14, and HRV3, which also lack pocket factors in native virions (54, 56, 63). There are 26 residues of VP1 that interact with pleconaril or WIN51711 (Fig. 3F and G and Fig. S6 and S13). Most of the interactions between VP1 and the inhibitors are hydrophobic (Fig. 3F and G and Fig. S13). Moreover, 4 hydrogen bonds connect pleconaril to VP1, providing specificity to the inhibitor-capsid interactions (Fig. S13). The GH loop from VP1 of EV-D68 becomes disordered when pleconaril binds to the pocket (35); a similar loss of the structure of the loop was not observed for EV70.

We compared the structures of WIN51711 and pleconaril in the VP1 pocket with already known structures of complexes of enteroviruses with these capsid-binding inhibitors. The structures of EV70, EV-D68, HRV14, and HRV16 in complex with pleconaril show that the isoxazole and trifluoromethyl ends of the molecule occupy equivalent spaces in the pockets of these viruses (Fig. S14). The only major difference is the dislocation of the phenoxy group in HRV14 (Fig. S14c). This is caused by the different shape of the pocket due to Tyr at a position homologous to Ile124 in EV70 (Fig. S14e). The change in the pleconaril structure is enabled by the flexible aliphatic linker between the isoxazole and phenoxy groups. Similarly, for WIN51711, the positions of the isoxazole and oxazoline groups inside the pocket remain conserved among the already known structures of enteroviruses complexed with this inhibitor (HRV14, Coxsackievirus A9 (CVA9), and poliovirus type 3 (Polio3)). In contrast, the positions of the isoxazole and oxazoline groups are swapped in the EV71–WIN51711 complex (Fig. S14d). This may be due to the low resolution of the crystal structure where the orientation of the molecule was ambiguous and the one with the higher map correlation being selected (40). In the EV70–WIN51711 complex, the aliphatic linker and phenoxy group are dislocated relative to those of other studied enteroviruses. This is again caused by differences in the shape of the pocket due to aromatic residues in a position homologous to Ile124 in EV70 (Fig. S14f). For both inhibitors, the flexibility of the aliphatic linker enables the binding of the inhibitors to enterovirus pockets with different shapes. Complexes of enteroviruses with pleconaril and WIN51711 contain only a few conserved interactions involving aromatic residues. In all these viruses, residues Trp98 and Tyr152 (EV70 numbering) are in proximity to the phenoxy group, whereas Phe120 and Tyr198 are in proximity to the isoxazole group (Fig. S14g and h). Although there are minor variations in the distances and positions of the groups, it is likely that stacking interactions play a major role in VP1-inhibitor complex stabilization. The alignment of homologous structures of 30 selected enteroviruses shows that these aromatic residues are highly conserved (Fig. 2C and Fig. S8), thus explaining the broad activity of WIN51711 and pleconaril against enteroviruses (50, 53).

It is challenging to predict the binding of putative pocket-targeting compounds to enteroviruses that lack the pocket factors in their capsids because the binding of natural pocket factors or artificial inhibitors induces conformational changes in the capsid protein VP1. Therefore, the structure of the virion of EV70 with empty VP1 pockets does not provide complete information for the *in silico* design of capsid-binding inhibitors. In contrast, the structures of EV70 in complex with WIN51711 and pleconaril enable the development of more efficient molecules.

## MATERIALS AND METHODS

### Virus propagation and purification.

EV70 (strain J670/71; ATCC VR-836) was propagated in human retina epithelial cells immortalized with human telomerase reverse transcriptase (hTERT RPE1; ATCC CRL-4000) cultivated in Dulbecco’s modified Eagle’s medium (DMEM)–F-12 medium enriched with 10% fetal bovine serum (FBS). For virus preparation, 100 pieces of tissue culture dishes with a diameter of 150 mm hTERT RPE1 cells grown to 90% confluence were infected with EV70 at 0.1 PFU per cell. The infection was allowed to proceed for 24 h at 33°C, at which point more than 90% of the cells exhibited a cytopathic effect. The supernatant was collected, and any remaining attached cells were removed from the dishes using cell scrapers. The supernatant was centrifuged at 10,000 rpm for 30 min in a Beckman Coulter Allegra 25R centrifuge with an A-10 rotor at 4°C. The resulting pellet was resuspended in 10 mL of resuspension buffer (0.25 M HEPES [pH 7.0], 0.25 M NaCl). This fraction was subjected to three rounds of freeze-thawing by sequential transfer between −80°C and 37°C and homogenized with a Dounce tissue grinder to break up the remaining cells. Cell debris was separated from the supernatant by centrifugation at 10,000 rpm for 30 min in a Beckman Coulter Allegra 25R centrifuge with an A-10 rotor at 4°C. The resulting supernatant was combined with the one obtained during the first low-speed centrifugation step. Virus particles were precipitated by adding polyethylene glycol 8000 (PEG 8000) and NaCl to final concentrations of 5% (wt/vol) and 0.5 M, respectively. The resulting solution was incubated at 4°C with shaking at 80 rpm overnight. The following day, the solution was spun down at 10,000 rpm for 30 min in a Beckman Coulter Allegra 25R centrifuge with an A-10 rotor at 4°C. The clearly visible white precipitate was resuspended in 12 mL of the resuspension buffer. MgCl_2_ was added to a final concentration of 5 mM, and the sample was subjected to DNase and RNase (final concentrations of 10 μg/mL each) treatment for 30 min at ambient temperature. Subsequently, trypsin was added to a final concentration of 80 μg/mL, and the mixture was incubated at 37°C for 15 min. EDTA (pH 9.5) was added to a final concentration of 15 mM, and a nonionic detergent, NP-40 (Sigma-Aldrich Inc.), was added to a final concentration of 1% (vol/vol). The solution was centrifuged at 4,500 rpm in a Beckman Coulter Allegra 25R centrifuge with an A-10 rotor at 4°C. The resulting supernatant was spun down through a 30% (wt/vol) sucrose cushion in resuspension buffer at 200,000 × *g* using a Beckman Coulter Ti50.2 rotor at 10°C. The pellet was resuspended in 1 mL of cold resuspension buffer and loaded onto a 60% (wt/wt) CsCl solution in resuspension buffer (0.25 M HEPES [pH 7.0], 0.25 M NaCl). After 24 h of incubation at ambient temperature, gradient ultracentrifugation was allowed to proceed for at least 12 h at 100,000 × *g* in a Beckman Coulter SW41Ti rotor. The opaque band containing the virus was extracted with an 18-gauge needle attached to a 3-mL disposable syringe. Finally, the virus was buffer exchanged into the resuspension buffer with a centrifugal filter device with a 100-kDa-molecular-weight cutoff (Corning Costar Spin-X) and concentrated to a final concentration of ~1 mg/mL.

### Cryo-electron microscopy and data collection.

A solution of EV70 particles (1 mg/mL) was applied to Quantifoil R2/1 300-mesh or C-flat 200-mesh holey carbon grids coated with a thin layer of supporting carbon film. The thin layer of carbon over the holes helped to adhere the virus better, thus increasing the density of the particles inside the holes. Vitrification was done using a Vitrobot Mark IV instrument by rapid plunging into liquid ethane. For the virus-inhibitor complex, the purified virus solution at a concentration of ~1 mg/mL was mixed and incubated for 30 min at 4°C with the capsid-binding inhibitor WIN51711 or pleconaril at a final concentration of 100 μg/mL. The virus–inhibitor complex was vitrified as described above.

Data were collected using an FEI Titan Krios transmission electron microscope operated at 300 kV. The sample in the column of the microscope was kept at −196°C. Images were recorded with an FEI Falcon II or Falcon III direct electron detection camera under low-dose conditions (see Table S1 in the supplemental material), with defocus values ranging from 1 to 3.0 μm at a nominal magnification of ×75,000. This setting resulted in a pixel size of 1.063 Å. Each image was recorded in movie mode with a 1-s total acquisition time and saved as a separate movie frame (see Table S1 for details).

### Single-particle data acquisition and image processing.

The acquired movie frames were motion corrected using MotionCor2 (71), and the defocus was estimated using Gctf (72). Particles in the motion-corrected micrographs were automatically picked using crYOLO software (73). The particles were extracted, 4× binned from the micrographs, and subjected to several rounds of two-dimensional (2D) classification in RELION. These steps removed the majority of the false-positive autopicked particles. An initial model with imposed icosahedral symmetry was generated using the binned particles and the stochastic gradient descent method (74) performed in RELION. The particles from selected classes were reextracted without binning, and using the rescaled initial model, the RELION 3dautorefine method was employed to perform 3D refinement according to the gold-standard procedure. The resulting offsets and orientations of the particles were used in the subsequent 3D classification step to further homogenize the particles. After further 3D refinement in RELION, the anisotropic magnification in the images was estimated using relion_ctf_refine, and beam-tilt was refined (75). Each acquisition area per hole was considered a separate optics group. Third- and fourth-order aberrations were estimated using relion_ctf_refine (76). This was followed by 3D refinement and iterated by alternating ctf refinement and 3D refinement until no improvement in resolution was achieved. Finally, particle polishing (75) was performed, followed by a final 3D refinement step. The Ewald sphere correction implemented in RELION (77) was performed on the refinements. During all refinement, 3D classification, and Ewald sphere correction steps, icosahedral symmetry was imposed on the model. The final maps were masked with a threshold mask and B-factor sharpened. To avoid overmasking, the phase-randomized masked half-map Fourier shell correlation (FSC) curve was carefully inspected (78), and the mask was visually inspected to avoid removing the protein densities in the final map. The resolution of the maps was estimated using the FSC_0.143_ threshold criterion (Fig. S8). Detailed workflows of the reconstruction of the EV70 native particles, EV70 altered particles, EV70 empty particles, EV70 with WIN51711, and EV70 with pleconaril are shown in Fig. S15 and S16 in the supplemental material.

### Model building and molecular docking.

Initially, a molecular model of the closest homologue, EV-D68 (Protein Data Bank [PDB] accession number 4WM8), was rigid-body fitted to the density map of the newly reconstructed EV70 using UCSF Chimera (79). The side chains of the amino acids were mutated and refitted in Coot (80), as were the loops not resolved in the EV-D68 structure being remodeled *de novo*. For inhibitor-containing virus particles, the initial position, orientation, and conformation of the inhibitor were transferred from an already solved model of EV-D68 (pleconaril) (PDB accession number 4WM7) and EV71 (WIN51711) (PDB accession number 3ZFG). Alternative orientations of the inhibitors (e.g., rotated 180°) were tested, and the model most representing the measured density was further refined. For refinement, cif files from the REFMAC (CCP4 v7.1) library were used. The models were refined in real-space using Phenix (81), and in reciprocal space using REFMAC 5 (82). The quality of the models was evaluated using MolProbity (83) and visually inspected after each refinement step in Coot, and finally, the model to map FSC was calculated to evaluate for model overfitting (Fig. S17). Figures of molecules were generated in ChimeraX (84). The final models were deposited in the PDB.

### Estimation of EV70 infectivity using plaque assays.

To estimate the infectivity and titer of EV70, a plaque assay using hTERT RPE1 cells cultivated in DMEM–F-12 medium plus 10% FBS at 37°C with 5% CO_2_ was performed. Cells were seeded into 6-well plates at a density of 5 × 10^5^ cells/well and left to adhere overnight. Adherent cells were infected with EV70 (400 μL of viral inoculum/well). After 2 h of incubation, the virus was discarded, and a mixture of fresh cultivation medium with 10% FBS and 2% low-melting-point agarose was added. Plates were incubated for 30 min at 4°C with 5% CO_2_. Plaques were stained with 0.2% trypan blue and fixed with a 3:1 mixture of methanol-acetic acid. Agarose was removed from the wells under tap water, and the number of plaques was counted. The experiments were carried out in triplicates.

To estimate the decrease in infectivity caused by a capsid-binding inhibitor, the virus inoculum (62.5 PFU/mL) was mixed with WIN51711 or pleconaril at various concentrations (final concentrations of 2, 1, 0.5, 0.25, 0.125, 0.0625, and 0.03125 μg/mL) and incubated for 30 min at 4°C. Maximum infectivity was represented by the virus without the inhibitor added. The decrease in virus infectivity was evaluated by a plaque assay. EC_50_ values were calculated from sigmoidal infectivity inhibition curves constructed by fitting the Hill equation to the measured data.

### Evaluation of the thermal stability of the EV70 particles.

Virions of EV70 at a concentration of 16 μg/mL in buffer containing 0.25 M HEPES (pH 7.0) and 0.25 M NaCl were incubated with SYBR green II (diluted 3,000 times from the stock solution according to the manufacturer’s instructions) in a total volume of 25 μL. The mixture was heated from 25°C to 68°C in 1°C increments, with a 2-min incubation time for each step, in a real-time PCR instrument (TOptical thermocycler; Biometra). The readout of fluorescence was performed at 25°C for each step. The fluorescence signal increases as the dye interacts with RNA that is released from thermally destabilized particles, or the dye might be able to enter structurally relaxed particles. The thermal stability of the virus was estimated as the temperature at which 50% of the maximal fluorescence value was achieved. The measurements were carried out in triplicates.

When testing the stability of EV70 with the capsid-binding inhibitor WIN51711 or pleconaril, virions (final concentration of 160 μg/mL) were incubated with the inhibitor (final concentration of 10 μg/mL) for 30 min at 4°C, followed by 10-fold dilution with buffer containing SYBR green II. The molar excess of the inhibitor per binding site was 15-fold.

### Data availability.

Cryo-EM electron density maps of the native EV70 virion, the altered particle, the empty particle, and the EV70-pleconaril and EV70-WIN51711 complexes have been deposited in the Electron Microscopy Data Bank (https://www.ebi.ac.uk/pdbe/emdb/) (accession numbers EMD-13022, EMD-13125, EMD-13126, EMD-13127, and EMD-13128), and the fitted coordinates have been deposited in the Protein Data Bank (https://www.rcsb.org) (PDB accession numbers 7OPX, 7OZI, 7OZJ, 7OZK, and 7OZL, respectively). Additional data that support the findings of this study are available from the corresponding author upon request.
