# Supplementary material for: Structure of Human Enterovirus 70 and Its Inhibition by Capsid-Binding Compounds
Source: J Virol. 2022 Aug 8;96(17):e00604-22. doi: 10.1128/jvi.00604-22 (PMC9472761; doi:10.1128/jvi.00604-22)
Supplement: Supplemental file 1 — Fig. S1 to S17; Tables S1 and S2. Download jvi.00604-22-s0001.pdf, PDF file, 7.9 MB [file jvi.00604-22-s0001.pdf]

Supplementary Information

Structure of human enterovirus 70 and its inhibition by capsid-binding inhibitors

Tibor Füzik, Jana Moravcová, Sergei Kalynych, Pavel Plevka

Corresponding author: Pavel Plevka; e-mail: pavel.plevka@ceitec.muni.cz

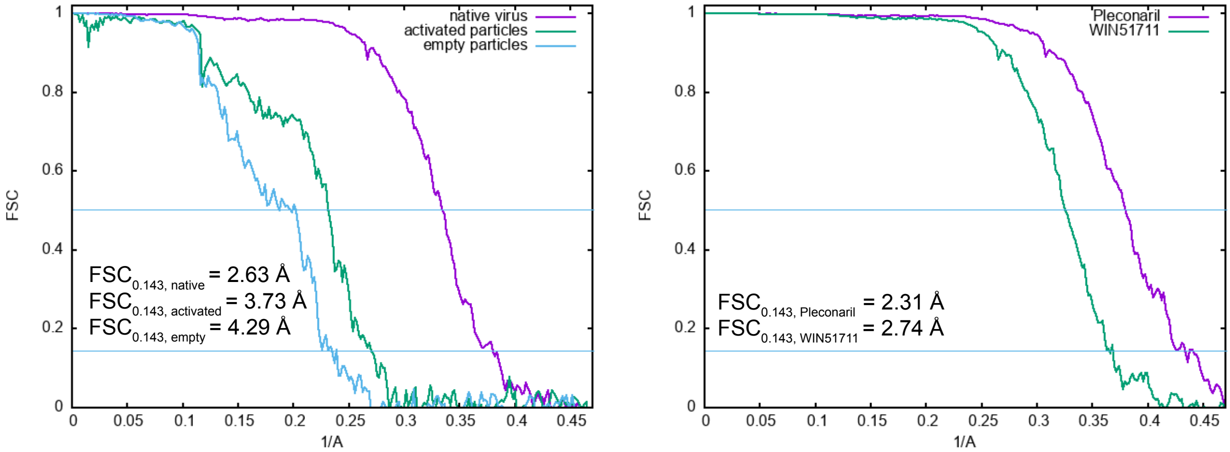

Fig. S1. Fourier shell correlation curves of half-maps from final gold-standard reconstructions. Resolutions were estimated according to the FSC 0.143 cut-off criterion.

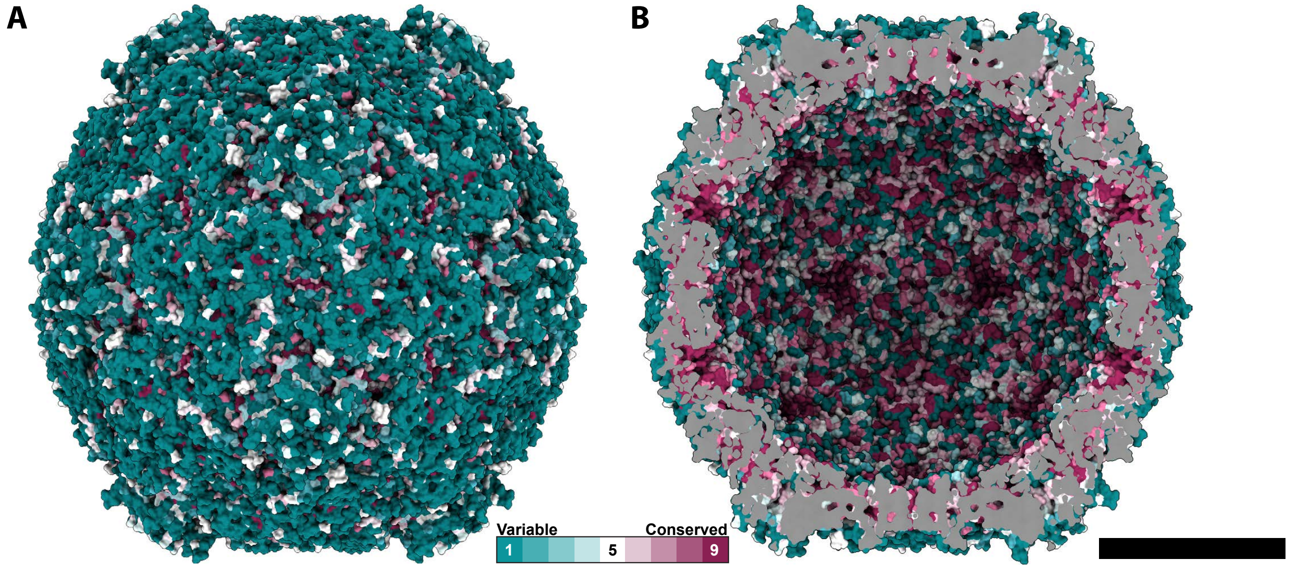

Fig. S2. Molecular surface of EV70 virion colored according to conservation of residues. Whole-particle (A) and particle with front half removed (B) are shown. The conservation of the amino acid sequence was evaluated for 30 enteroviruses (SFig. 3). Coloring was generated by Consurf server (https://consurf.tau.ac.il/) (1). The scale bar represents 10 nm.

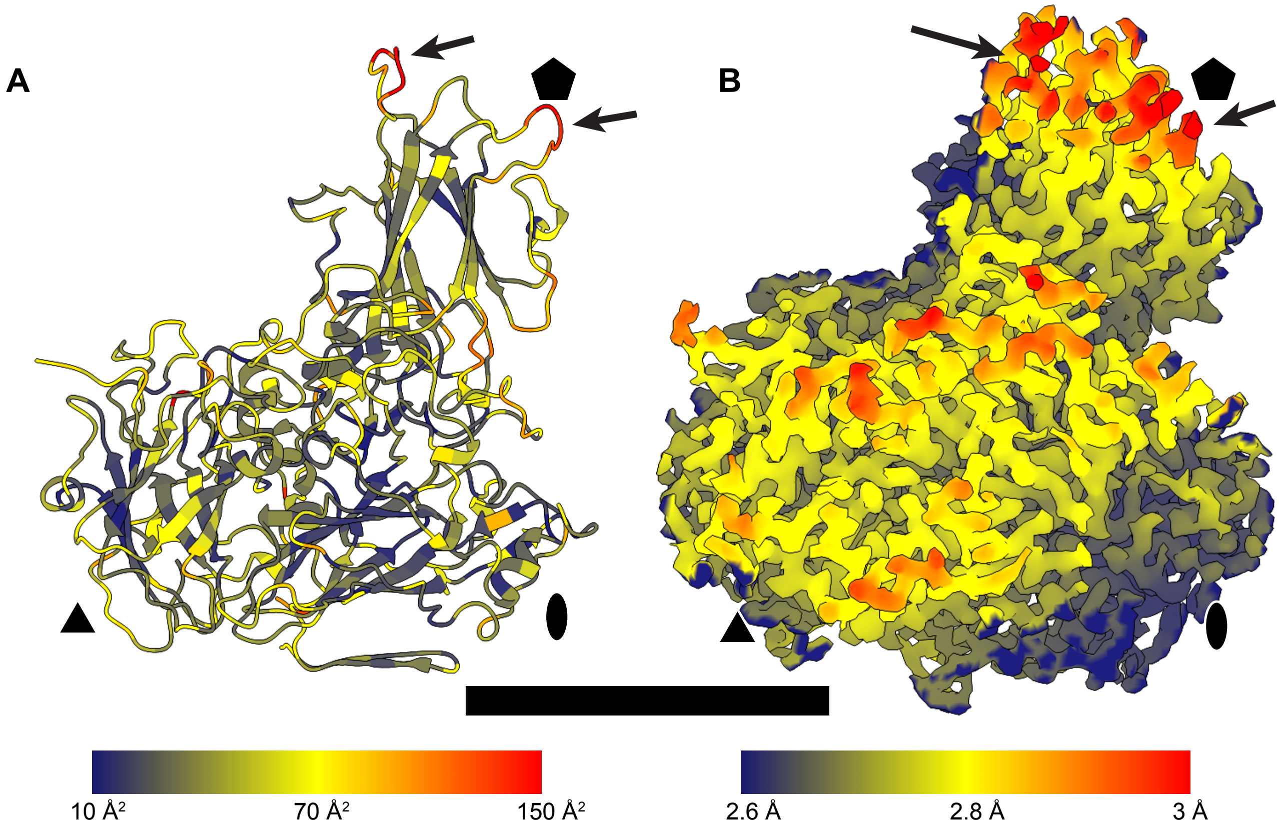

Fig. S3. Flexibility of protomer regions of EV70 native virion. Cartoon representation of EV70 protomer colored according to B-factors of atoms (A). Electrostatic potential density map cropped around EV70 protomer colored according to local map resolution (B). Arrows point to regions where VP1 BC and DE loops are located. Selected fivefold, threefold and twofold axes of symmetry are indicated with pentagon, triangle and oval respectively. The scale bar represents 5 nm.

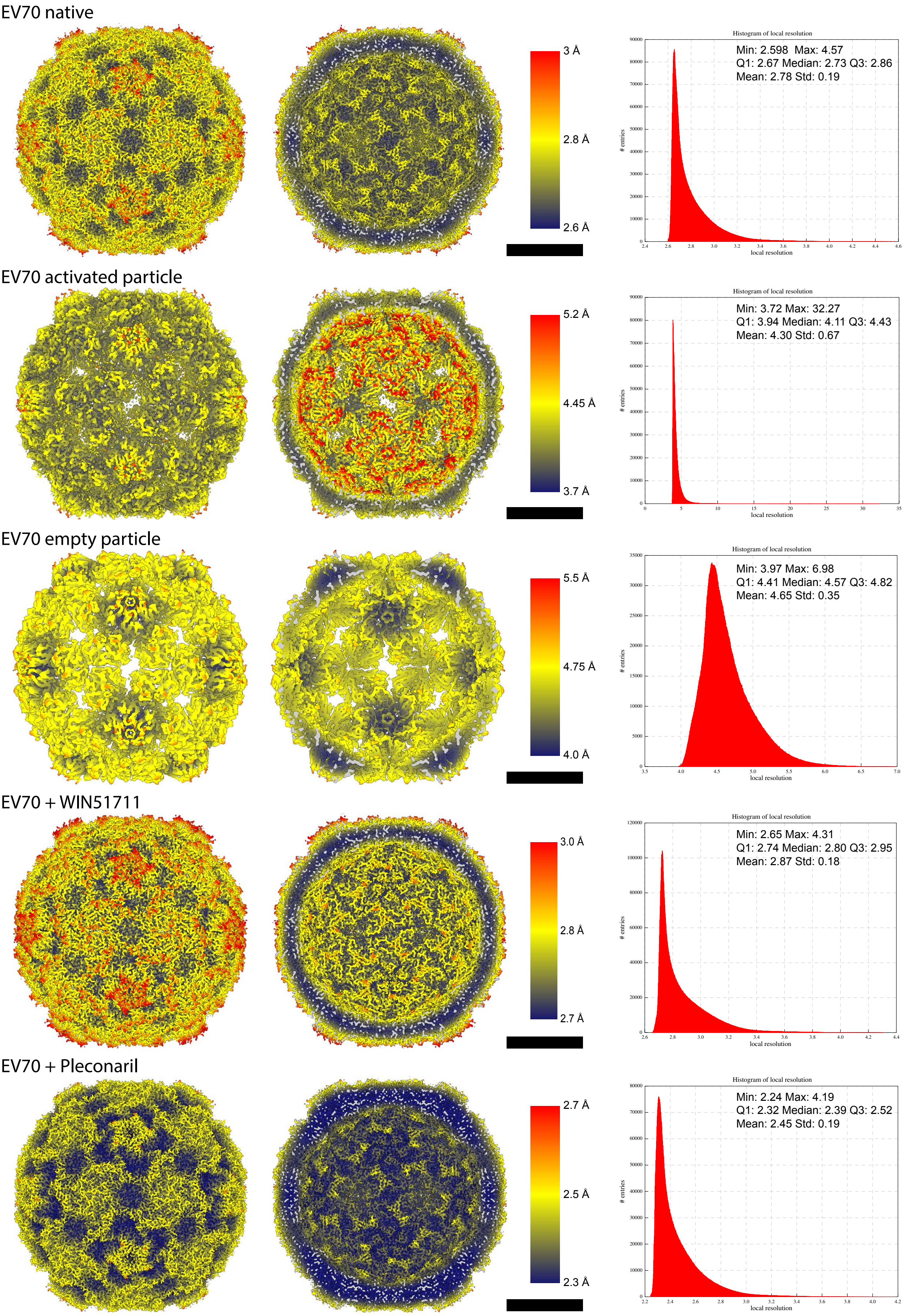

Fig. S4. Electrostatic potential surfaces colored according to local resolution of cryo-EM reconstructions. The outer surface of the particles is shown in the left column, and the central cut is shown in the middle column. Histogram plots show the distribution of the local resolution bins of the masked map (the mask applied around the protein shell). The scale bar represents 10 nm.

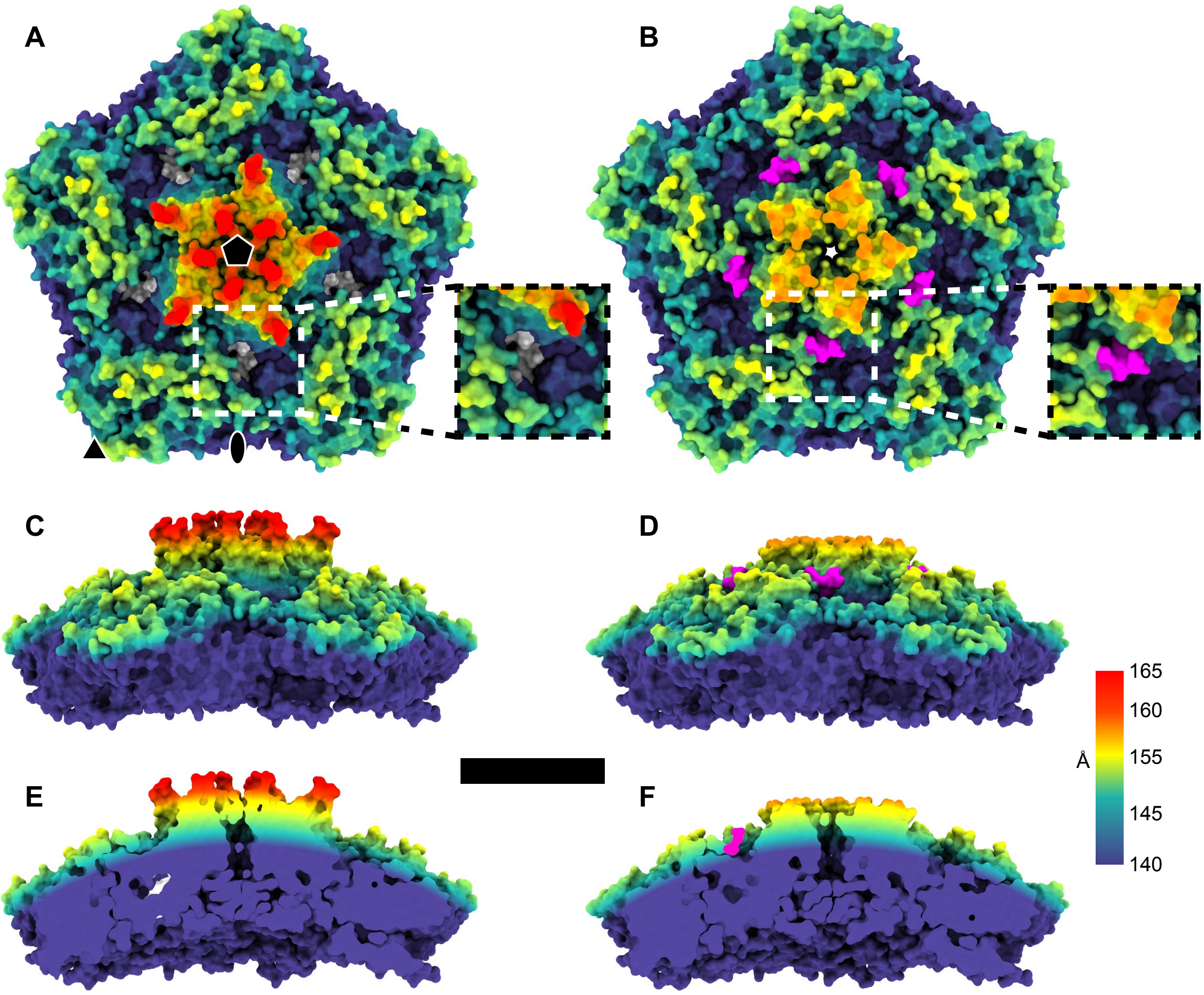

Fig. S5. Pentamer of capsid protein protomers of EV70 (A) and EV-D68 (pdb ID:4wm8) (B) showing canyon surrounding fivefold symmetry axis. EV70 lacks the alpha helical extension present at the C-terminus of VP3 in EV-D68 (highlighted in magenta on panels (B, D, F)). The entrance of the EV70 pocket is highlighted in white in panel (A, E). Side views of EV70 pentamer (C) and EV-D68 pentamer (D) are shown together with central sections of EV70 (E) and EV-D68 (F) pentamer side views. The molecular surfaces are colored according to the distance from the particle center. Selected fivefold, threefold and twofold axes of symmetry are indicated with pentagon, triangle and oval respectively. The scale bar represents 5 nm.

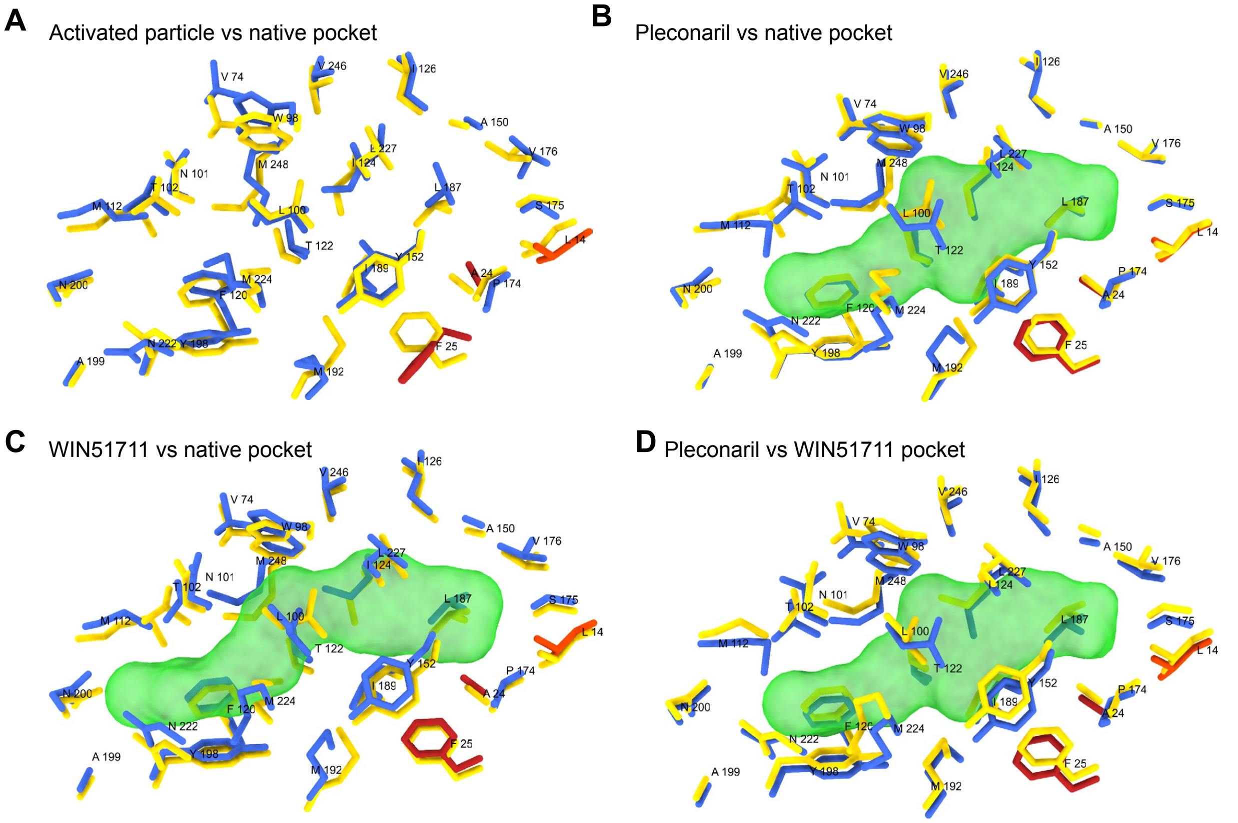

E

	Amino acid residue	Native vs Activated particle	Native vs Pleconaril	Native vs WIN51711	Pleconaril vs WIN51711
VP1	Val 74	4.14	0.24	0.2	0.18
VP1	Trp 98	13.80	0.57	0.55	0.23
VP1	Leu 100	11.50	0.56	0.93	0.76
VP1	Asn 101	9.57	0.56	0.60	0.49
VP1	Thr 102	9.00	0.41	0.62	0.28
VP1	Met 112	7.64	0.86	0.86	0.76
VP1	Phe 120	11.80	0.18	0.21	0.07
VP1	Thr 122	10.80	0.16	0.18	0.09
VP1	Ile 124	13.37	0.21	0.42	0.42
VP1	Ile 126	15.35	0.20	0.09	0.23
VP1	Ala 150	6.99	0.07	0.10	0.13
VP1	Tyr 152	11.58	0.21	0.71	0.69
VP1	Pro 174	7.17	0.22	0.13	0.28
VP1	Ser 175	9.03	0.14	0.11	0.14
VP1	Val 176	9.43	0.07	0.09	0.10
VP1	Leu 187	13.60	0.14	0.19	0.16
VP1	Ile 189	12.40	0.16	0.23	0.15
VP1	Met 192	10.82	0.71	0.77	0.17
VP1	Tyr 198	12.28	0.22	0.13	0.10
VP1	Ala 199	13.46	0.14	0.06	0.11
VP1	Asn 200	13.43	0.20	0.14	0.15
VP1	Asn 222	4.65	0.53	0.44	0.16
VP1	Met 224	4.39	1.18	1.08	0.53
VP1	Leu 227	3.09	0.26	0.28	0.12
VP1	Val 246	5.90	0.13	0.12	0.08
VP1	Met 248	6.18	0.43	0.30	0.42
VP3	Ala 24	9.62	0.09	0.12	0.10
VP3	Phe 25	10.44	0.18	0.17	0.27
VP3*	Leu 14	12.13	0.10	0.23	0.16
**Average**		9.78	0.31	0.35	0.26
**SD**		3.35	0.26	0.29	0.21

Fig. S6. Comparison of positions of side chains of pocket-forming residues of EV70 virion, EV70 in complex with pleconaril, and EV70 in complex with WIN51711. Coloring of the side chains: blue - VP1; red - VP3; orange - VP3 neighboring subunit; gold - sidechains belonging to the compared structure (panel A - WIN51711; panel B - native EV70; panel C - native EV70). Pleconaril (panel A, B) and WIN51711 (panel C) are shown as green semi-transparent molecular surfaces.

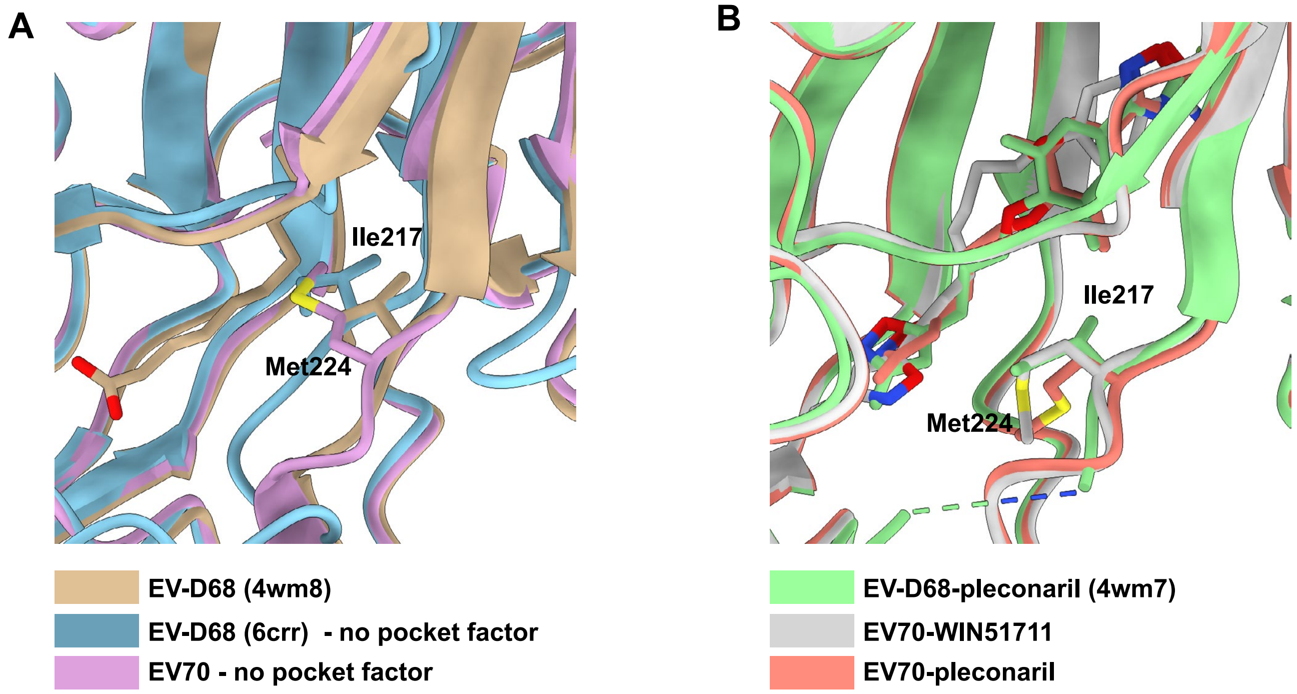

Fig. S7. Comparison of hydrophobic pocket of EV-D68 and EV70 with and without capsid-binding inhibitors. VP1 structures of EV-D68 and EV70 were structurally aligned to compare the structure of the hydrophobic pocket and to identify the homologous interacting amino acid residues in VP1. (A) X-tal (pdb ID:4wm8; (2)) and cryo-EM (pdb ID:6crr, (3)) structures of EV-D68 representing particles with and without pocket factor are compared with cryo-EM structure of EV70 without pocket factor. Amino acid residues partially blocking the hydrophobic pocket are shown. (B) Capsid-binding inhibitors bound inside hydrophobic pocket are shown. EV-D68–pleconaril (pdb ID:4wm7; (2)), EV70–WIN51711 and EV70–pleconaril are shown. Amino acid residues that blocked the hydrophobic pocket adopt an alternative conformation to allow the binding of the inhibitors.

VP1 1/3

```
                                α        α          α
                         pid 1                                                                                80
 1 P32537|EV70        100.0% ---LPE---QAATTQIGE----------IVKTVANTVESEIKAELGVIPSLNAVETGATSNTEPEEAIQTRTVINMHGTA
 2 Q68T42|EV68         65.9% SNHLHG---AEAAYQVES----------IIKTATDTVKSEINAELGVVPSLNAVETGATSNTEPEEAIQTRTVINQHGVS
 3 B9VUU3|EV71         35.9% ------RVADVIESSIGDSVSRALTQALPAPTGQNTQVSSHRLDTGKVPALQAAEIGASSNASDESMIETRCVLNSHSTA
 4 O91734|Echo1        38.5% ----------FYQGDVQNAVE------GAMVRVADTVQTS-ATNSERVPNLTAVETGHTSQAVPGDTMQTRHVINNHVRS
 5 Q9YLJ1|Echo5        38.9% ----------DLQGDSEHAVE------SAVSRVADTIMSG-PSNSQQVPALTAVETGHTSQVVPSDTIQTRHVQNFHSRS
 6 Q66474|Echo6        38.4% ----------FYQNDVQNAVE------RSIVRVADTLPSG-PSNSESIPALTAAETGHTSQVVPSDTIQTRHVRNFHVRS
 7 Q66577|Echo9        35.3% ----------ELQNDVRQAVE------GAIGRVADTIRSG-PSNSEAVPALTAAETGHTSQVVPSDTMQTRHVKNYHSRS
 8 P29813|Echo11       37.7% ----------LLQGDVVEAVE------NAVARVADTIGSG-PSNSQAVPALTAVETGHTSQVTPSDTMQTRHVKNYHSRS
 9 Q66575|Echo12       38.3% ---------QLLQGDVEEAVN------RAVARVADTLPTG-PRNSESIPALTAAETGHTSQVVPGDTMQTRHVKNYHSRT
10 Q66790|Echo16       39.6% -------------GEPGKAIE------SAISRVADTISSG-PTNSEQVPALTAAETGHTSQVVPGDTIQTRHVKNYHSRS
11 Q9WN78|Echo30       36.2% ----------LYQNDPEGALN------KAVGRVADTIASG-PVNTEQIPALTAVETGHTSQVVPSDTMQTRHVVNFHTRS
12 P21404|CoxA9        35.9% ----------KLQGDVEEAIE------RARCTVADTMRTG-PSNSASVPALTAVETGHTSQVTPSDTMQTRHVKNYHSRS
13 Q65900|CoxA16       36.2% ------GIADMIDQAVTSRVGRALTSLQVEPTAANTNASEHRLGTGLVPALQAAETGASSNAQDENLIETRCVLNHHSTQ
14 P22055|CoxA21       32.8% ------GIEDLIDTAIKNALRVSQPLRPSQLK-----QPN-GVNSQEVPALTAVETGASGQAIPSDVVETRHVINYKTRS
15 P36290|CoxA24       34.4% ------GIEETIDTVISNALQLSQPKPQKQLTAQSTPSTS-GVNSQEVPALTAVETGVSGQAIPSDVIETRHVVNYKTRS
16 P08291|CoxB1        36.9% ----------FYQGPVEESVE------RAMVRVADTVSSK-PTNSESIPALTAAETGHTSQVVPSDTMQTRHVKNYHSRS
17 Q9YLG5|CoxB2        38.2% ----------FYQSPVEESIE------RSIGRVADTIGSG-PSNSEAIPVLTAVETGHTSQVTPSDTMQTRHVHNYHSRS
18 P03313|CoxB3        37.2% ----------FFQGPVEDAIT------AAIGRVADTVGTG-PTNSEAIPALTAAETGHTSQVVPGDTMQTRHVKNYHSRS
19 Q86887|CoxB4        35.9% ----------FYQGPTEESVE------RAMGRVADTIARG-PSNSEQIPALTAVETGHTSQVDPSDTMQTRHVHNYHSRS
20 Q03053|CoxB5        39.1% ----------FYQGPPGEAVE------RAIARVADTISSG-PVNSESIPALTAAETGHTSQVVPADTMQTRHVKNYHSRS
21 Q9QL88|CoxB6        38.2% ----------FYQSPVEGAIE------RAIARVADTMPSG-PTNSEAVPALTAVETGHTSQVVPSDNMQTRHVKNYHSRS
22 P23008|HRV1A        37.7% ---------PIEQNPVENYIDE---VLNEVLVVPNIKESH-HTTSNSAPLLDAAETGHTSNVQPEDAIETRYVITSQTRD
23 P12916|HRV1B        36.7% ---------PIEQNPVENYIDE---VLNEVLVVPNIKESH-HTTSNSAPLLDAAETGHTSNVQPEDAIETRYVMTSQTRD
24 P04936|HRV2         35.9% ----------IAQNPVENYIDE---VLNEVLVVPNINSSN-PTTSNSAPALDAAETGHTSSVQPEDVIETRYVQTSQTRD
25 Q82081|HRV3         38.2% --ALTEGLSDELEEVIVEKTK----------QTLASVSSG-PKHTQSVPALTANETGATLPTRPSDNVETRTTYMHFNGS
26 P03303|HRV14        37.2% --ALTEGLGDELEEVIVEKTK----------QTVASISSG-PKHTQKVPILTANETGATMPVLPSDSIETRTTYMHFNGS
27 Q82122|HRV16        34.8% ---------PITQNPVERYVDE---VLNEVLVVPNINQSH-PTTSNAAPVLDAAETGHTNKIQPEDTIETRYVQSSQTLD
28 P03301|Polio1       32.5% ------GLGQMLESMIDNTVRETVGAATSRDALPNTEASG-PAHSKEIPALTAVETGATNPLVPSDTVQTRHVVQHRSRS
29 P06210|Polio2       32.8% ------GLGDLIEGVVEGVTRNALTPLTPANNLPDTQSSG-PAHSKETPALTAVETGATNPLVPSDTVQTRHVIQKRTRS
30 P03302|Polio3       31.6% ------GIEDLISEVAQGALT--LSLPKQQDSLPDTKASG-PAHSKEVPALTAVETGATNPLAPSDTVQTRHVVQRRSRS
   consensus/100%            ................t......................t...t.t.hP.LpAsEhGho..h.stp.hpTRhs....sht
   consensus/90%             ...........hpt..tt.hp...........h.sh..ot...potthPsLsAsETGtos.h.Pt-shpTRhVhp.psts
   consensus/80%             ..........hhpssltphlp......t.h.pssso.tot.sspoptlPuLsAsETGtTups.PpDshpTRaVhs.+opu
   consensus/70%             ..........hhpsslppslc......pthhplssThtSu.PssSpplPALTAsETGtTSplhPuDshpTRaVhNh+oRS

                               α           β                      β              α                β
                         pid 81                                                                               160
 1 P32537|EV70        100.0% ECLVENFLGRSALVCMRSFEYKNHSTS-TSSIQKNFFIWTLNTRELVQIRRKMELFTYLRFDTEITIVPTLRLFSSSNVS
 2 Q68T42|EV68         65.9% ETLVENFLGRAALVSKKSFEYKNHASS-SAGTHKNFFKWTINTKSFVQLRRKLELFTYLRFDAEITILTTVAVNGNNDST
 3 B9VUU3|EV71         35.9% ETTLDSFFSRAGLVGEIDLPLEGT------TNPNGYANWDIDITGYAQMRRKVELFTYMRFDAEFTFVACTP--------
 4 O91734|Echo1        38.5% ESTIENFLARSACVFYLEYKTGT-----K-EDSNSFNNWVITTRRVAQLRRKLEMFTYLRFDMEITVVITSSQDQSTS-Q
 5 Q9YLJ1|Echo5        38.9% ESTIENFLSRSACVHIANYNAKG-----DKTDVNRFDRWEINIREMVQLRRKCEMFTYLRFDIEVTFVITSKQDQGPK-L
 6 Q66474|Echo6        38.4% ESSVENFLSRSACVYIVEYKTQD-----T-TPDKMYDSWVINTRQVAQLRRKLEFFTYVRFDVEVTFVITSVQDDSTR-Q
 7 Q66577|Echo9        35.3% ESTIENFLCRSACVRMAKYEARG-----DPESTDRFDAWEISVRDMVQMRRKCEMFTYLRFDVEVTFVITSYQHQGS--I
 8 P29813|Echo11       37.7% ESSIENFLSRSACVYMGGYHTTN-----T-DQTKLFASWTISARRMVQMRRKLEIFTYVRFDVEVTFVITSKQDQGSR-L
 9 Q66575|Echo12       38.3% ESSVENFLCRAACVYITKYKTKD-----S-DPVQRYANWRINTRQMVQLRRKFELFTYLRFDMEVTFVITSSQDDGTQ-L
10 Q66790|Echo16       39.6% ESTIENFLCRSACVHIARYEAGA-----NASNEDRFVRWEINNKELVQLRRKCEMFTYLRYDVEVTFVITSQQDQGTD-L
11 Q9WN78|Echo30       36.2% ESSLENFMGRAACAYIAHYTTEK-----ANDDLDRYTNWEITTRQVAQLRRKLEMFTYMRFDLEITFVITSSQRTSNR-Y
12 P21404|CoxA9        35.9% ESTVENFLGRSACVYMEEYKTTD-----K-HVNKKFVAWPINTKQMVQMRRKLEMFTYLRFDMEVTFVITSRQDPGTT-L
13 Q65900|CoxA16       36.2% ETTIGNFFSRAGLVSIITMPTTGT------QNTDGYVNWDIDLMGYAQMRRKCELFTYMRFDAEFTFVAAKP--------
14 P22055|CoxA21       32.8% ESCLESFFGRAACVTILSLTNSS--K--SGEEKKHFNIWNITYTDTVQLRRKLEFFTYSRFDLEMTFVFTENYPSTA---
15 P36290|CoxA24       34.4% ESTLESFFGRSACVTMLEVENFNATT--EADKKKQFTTWAITYTDTVQLRRKLEFFTYSRFDLEMTFVITERYYTSN---
16 P08291|CoxB1        36.9% ESSIENFLCRSACVYYATYNNNS-------EK--GYAEWVINTRQVAQLLRRKLEFTYLRFDLELTFVITSAQEPSTA-T
17 Q9YLG5|CoxB2        38.2% ESSVENFLARSACVFYTTYTNSK-----NAAKEKKFATWKVSVRQAAQLRRKLELFTYLRCDIELTFVITSAQDPSTA-T
18 P03313|CoxB3        37.2% ESTIENFLCRSACVYFTEYKNSG-------AK--RYAEWVLTPRQAAQLRRKLEFFTYVRFDLELTFVITSTQQPSTT-Q
19 Q86887|CoxB4        35.9% ESSIENFLCRSACVIYIKYSSAE-----S-NNLKRYAEWVINTRQVAQLRRKMEMFTYIRCDMEQTFVITSHQEMSTA-T
20 Q03053|CoxB5        39.1% ESTVENFLCRSACVYYTTYKNHG-------TDGDNFAYWVINTRQVAQLRRKLEMFTYARFDLELTFVITSTQEQSTI-Q
21 Q9QL88|CoxB6        38.2% ETSVENFLCRSACVYFTTYKNQT-------GATNRFASWVITTRQVAQLRRKLEMFTYLRFDIELTFVITSAQDQSTI--
22 P23008|HRV1A        37.7% EMSIESFLGRSGCVHISRIKVDYTD---YNGQDINFTKWKITLQEMAQIRRKFELFTYVRFDSEITLVPCIA-----G-R
23 P12916|HRV1B        36.7% EMSIESFLGRSGCVHISRIKVDYND---YNGVNKNFTTWKITLQEMAQIRRKFELFTYVRFDSEVTLVPCIA-----G-R
24 P04936|HRV2         35.9% EMSLESFLGRSGCIHESKLEVTLAN---YNK--ENFTVWAINLQEMAQIRRKFELFTYTRFDSEITLVPCIS-----A-L
25 Q82081|HRV3         38.2% ETDVESFLGRAACVHVTEIKNKNAAGLDNHRKEGLFNDWKINLSSLVQLRKKLELFTYVRFDSEYTILATASQPEASS--
26 P03303|HRV14        37.2% ETDVECFLGRAACVHVTEIQNKDATGIDNHREAKLFNDWKINLSSLVQLRKKLELFTYVRFDSEYTILATASQPDSAN--
27 Q82122|HRV16        34.8% EMSVESFLGRSGCIHESVLDIV-DN---Y--NDQSFTKWNINLQEMAQIRRKFEMFTYARFDSEITMVPSVA-----A-K
28 P03301|Polio1       32.5% ESSIESFFARGACVAIITVDNSASTK----NKDKLFTVWKITYKDTVQLRRKLEFFTYSRFDMEFTFVVTANFTETN---
29 P06210|Polio2       32.8% ESTVESFFARGACVAIIEVDNDAPTK----RASKLFSVWKITYKDTVQLRRKLEFFTYSRFDMEFTFVVTSNYTDAN---
30 P03302|Polio3       31.6% ESTIESFFARGACVAIIEVDNEQPTT----RAQKLFAMWRITYKDTVQLRRKLEFFTYSRFDMEFTFVVTANFTNAN---
   consensus/100%            E..ltsFhsRuuhh....h...............a..W.ls..thsQhh++h..FTY.RhD.E.Thl.s..........
   consensus/90%             E.slEsFhsRuuhVhh.php.t........t..t.as.W.IshpphsQhRRKhEhFTY.RFD.EhThV.s..........
   consensus/80%             EoolEsFhsRuACVhhhphpspt.......tt.p.as.W.IshpphsQlRRKhEhFTYhRFDhEhThVhTtt...ss...
   consensus/70%             EoolEsFLuRuACVhhhphcsps.......pptptFspWtIshpphsQlRRKhEhFTYhRFDhEhTFVhTss...us...
```

Fig. S8. Multiple sequence alignment of capsid proteins of selected enteroviruses. VP1, VP2, VP3 and VP4 protein sequences were aligned using the Clustal Omega tool (4) and viewed using mView (5). Secondary structure elements and structural features according to position in EV70 are shown by color bars over the sequences. Color coding of the elements: yellow - alpha helices; green - beta sheets; black - loops; magenta - pocket forming amino acids; cyan - VP1 BC- and DE-loop. *(Continued on next page....)*

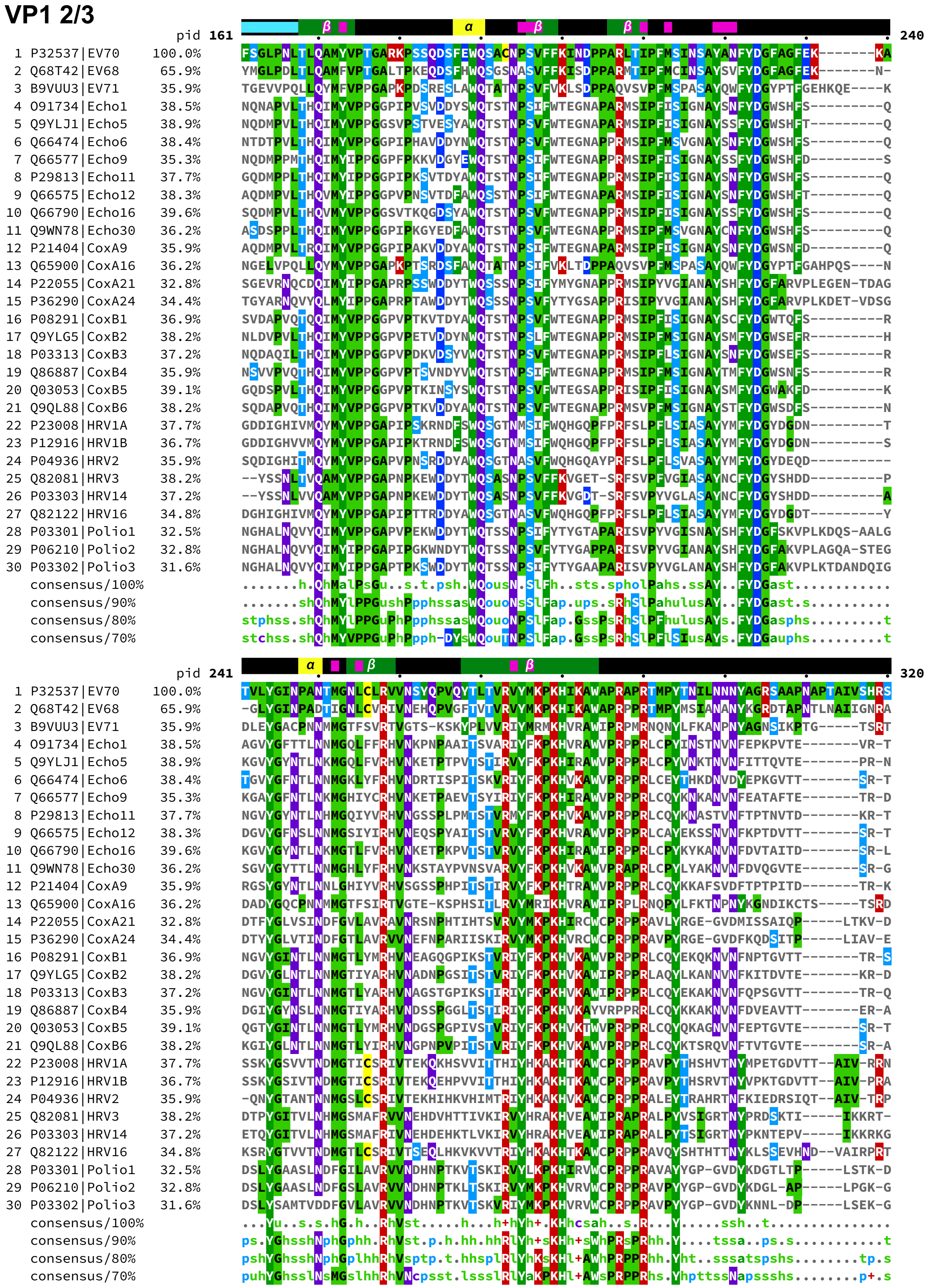

Fig. S8. Multiple sequence alignment of capsid proteins of selected enteroviruses. VP1, VP2, VP3 and VP4 protein sequences were aligned using the Clustal Omega tool (4) and viewed using mView (5). Secondary structure elements and structural features according to position in EV70 are shown by color bars over the sequences. Color coding of the elements: yellow - alpha helices; green - beta sheets; black - loops; magenta - pocket forming amino acids; cyan - VP1 BC- and DE-loop. *(Continued on next page....)*

VP1 3/3

```
                                 pid 321                 .          .    ] 344
 1 P32537|EV70     100.0%        TIKTMPNDINLTTA----------
 2 Q68T42|EV68      65.9%        SVTTMPHNIVTT------------
 3 B9VUU3|EV71      35.9%        AITTL-------------------
 4 O91734|Echo1     38.5%        NIITT-------------------
 5 Q9YLJ1|Echo5     38.9%        DLNDVPKSEHNMHTY---------
 6 Q66474|Echo6     38.4%        SITITNSKH-METH----------
 7 Q66577|Echo9     35.3%        TINTVPVSNHGSGRRGDLAALSTH
 8 P29813|Echo11    37.7%        SINYIPETV-KPDL----SNY---
 9 Q66575|Echo12    38.3%        SITEVPSLR-PSVVNT--------
10 Q66790|Echo16    39.6%        TITTVPQVEHNLRTA---------
11 Q9WN78|Echo30    36.2%        KITLDRSTH-NPVLTT--------
12 P21404|CoxA9     35.9%        DINTVTTVA-QSRRRGDMSTLNTH
13 Q65900|CoxA16    36.2%        KITTL-------------------
14 P22055|CoxA21    32.8%        SITTF-------------------
15 P36290|CoxA24    34.4%        NINTF-------------------
16 P08291|CoxB1     36.9%        NITTT-------------------
17 Q9YLG5|CoxB2     38.2%        SLTTT-------------------
18 P03313|CoxB3     37.2%        SITTMTNT----------------
19 Q86887|CoxB4     35.9%        SLVTT-------------------
20 Q03053|CoxB5     39.1%        EITAMQTT----------------
21 Q9QL88|CoxB6     38.2%        NITTMNTT----------------
22 P23008|HRV1A     37.7%        TITTA-------------------
23 P12916|HRV1B     36.7%        SMKTV-------------------
24 P04936|HRV2      35.9%        IITTA-------------------
25 Q82081|HRV3      38.2%        NIKTY-------------------
26 P03303|HRV14     37.2%        DIKSY-------------------
27 Q82122|HRV16     34.8%        NLTTV-------------------
28 P03301|Polio1    32.5%        L-TTY-------------------
29 P06210|Polio2    32.8%        L-TTY-------------------
30 P03302|Polio3    31.6%        L-TTY-------------------
   consensus/100%                ........................
   consensus/90%                 .hpth...................
   consensus/80%                 plsoh...................
   consensus/70%                 sIsTh...................
```

Fig. S8. Multiple sequence alignment of capsid proteins of selected enteroviruses. VP1, VP2, VP3 and VP4 protein sequences were aligned using the Clustal Omega tool (4) and viewed using mView (5). Secondary structure elements and structural features according to position in EV70 are shown by color bars over the sequences. Color coding of the elements: yellow - alpha helices; green - beta sheets; black - loops; magenta - pocket forming amino acids; cyan - VP1 BC- and DE-loop. *(Continued on next page....)*

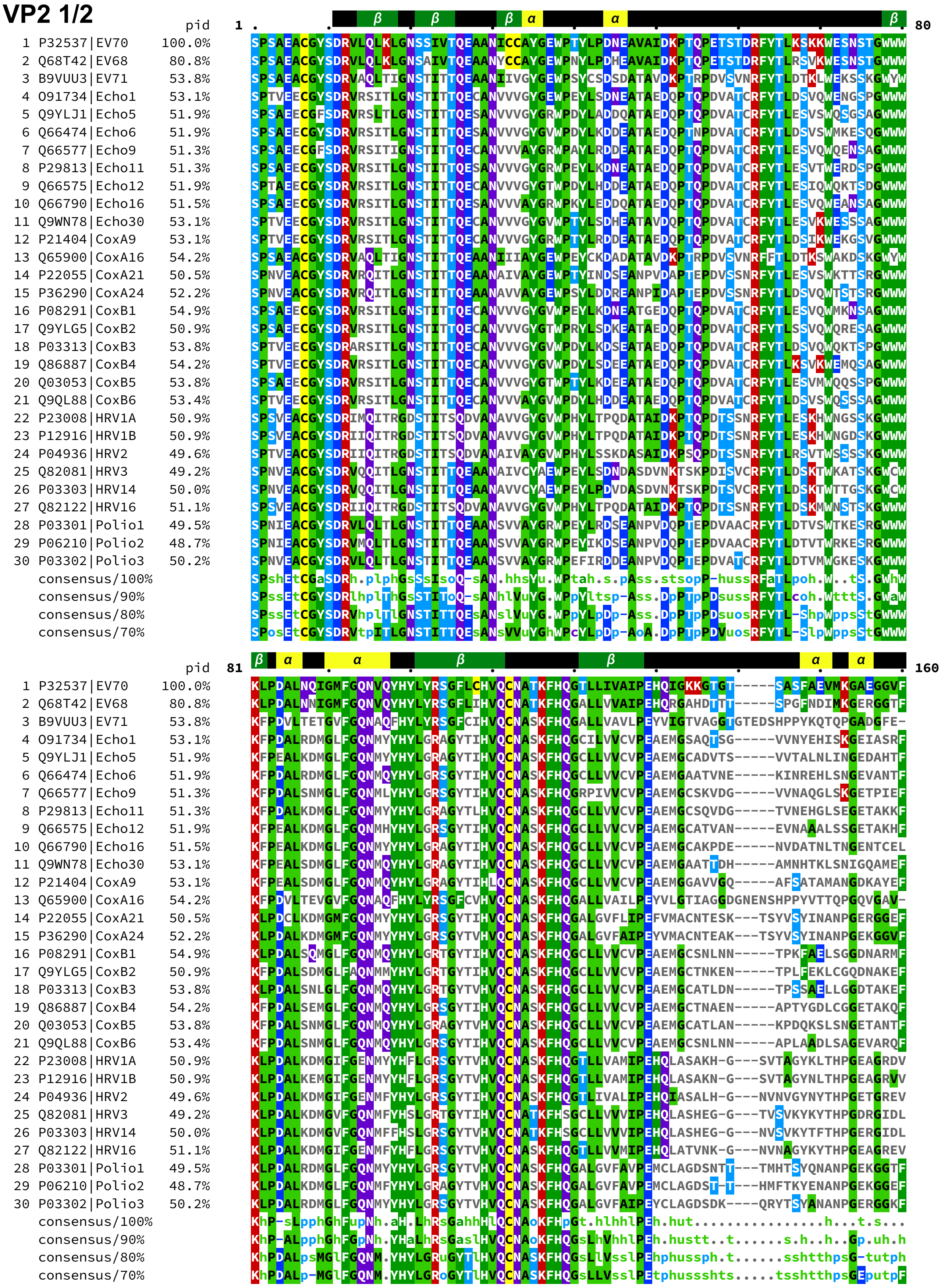

Fig. S8. Multiple sequence alignment of capsid proteins of selected enteroviruses. VP1, VP2, VP3 and VP4 protein sequences were aligned using the Clustal Omega tool (4) and viewed using mView (5). Secondary structure elements and structural features according to position in EV70 are shown by color bars over the sequences. Color coding of the elements: yellow - alpha helices; green - beta sheets; black - loops. *(Continued on next page....)*

VP2 2/2

```
                        pid 161  α       .         .  α    . α     .         .         .         .        . 240
 1 P32537|EV70    100.0%      EQPY------------------LLDDGTSLACALVYPHQWINLRTNNSATIVLPWMNSAPMDFALRHNNWTLAVIPVCPL
 2 Q68T42|EV68     80.8%      NHPY------------------VLDDGTSIACATIFPHQWINLRTNNSATIVLPWMNVAPMDFPLRHNQWTLAVIPVVPL
 3 B9VUU3|EV71     53.8%      -----------------LQHPYVLDAGIPISQLTVCPHQWINLRTNNCATIIVPYINALPFDSALNHCNFGLLVVPISPL
 4 O91734|Echo1    53.1%      TTTT------TAEDHGVQAAVWNAGMGVGVGNLTIFPHQWINLRTNNSATIVMPYVNSVPMDNMYRHHNFTLMIIPFVPL
 5 Q9YLJ1|Echo5    51.9%      SPSEA-----TAEAGKVQTAVCNAGMGVAVGNLTIFPHQWINLRTNNCATIVMPYINSVPMDNMFRHYNFTLMVIPFAPL
 6 Q66474|Echo6    51.9%      TGTK------SSNTNGVQQAVFNAGMGVRVGNLTVFPHQWINLRTNNCATIVMPYINSVPMDNMFRHYNFTLMIIPFAKL
 7 Q66577|Echo9    51.3%      ERTST-----TAEVGVVQKAVYNAGMGVGVGNLTIFPHQWINLRRNNSATIVIPYINCVPMDNMFRHNNFTLMVILFAPL
 8 P29813|Echo11   51.3%      SSTS------TNGTNTVQTIVTNAGMGVGVGNLTIYPHQWINLRTNNCATIVMPYINNVPMDNMFRHHNFTLMIIPFVPL
 9 Q66575|Echo12   51.9%      AKTG------ATGTHTVQSIVTNAGMGVGVGNLTIFPHQWINLRTNNSATIVMPYINSVPMDNMFRHYNFTLMIIPFVPL
10 Q66790|Echo16   51.5%      TAGAA-----PAEKGKVQTAVCNATMGVAVGNLTIFPHQWINLRTNNCATIVMPYINSVPMDNMFRHYNFTLMVIPFVPL
11 Q9WN78|Echo30   53.1%      SAGK------STDQTGPQTAVHNAGMGVAVGNLTIYPHQWINLRTNNSATIVMPYINSVPMDNMYRHYNFTLMVIPFAKL
12 P21404|CoxA9    53.1%      TSAT------QSDQTKVQTAIHNAGMGVGVGNLTIYPHQWINLRTNNSATIVMPYINSVPMDNMFRHYNFTLMVIPFVKL
13 Q65900|CoxA16   54.2%      -----------------LTNPYVLDAGVPLSQLTVCPHQWINLRTNNCATIIVPYMNTVPFDSALNHCNFGLIVVPVVPL
14 P22055|CoxA21   50.5%      TNTYNPSNTDVSEGRQFAALDYLLGSGVLAGNAFVYPHQIINLRTNNSATIVVPYVNSLVIDCMAKHNNWGIVILPLAPL
15 P36290|CoxA24   52.2%      SSTYNPSE-EASEGRKFAALDYLLGCGVLAGNAFVYPHQIINLRTNNSATLVLPYVNSLAIDCMAKHNNWGLVILPLCKL
16 P08291|CoxB1    54.9%      TDTE-VG---TSNDKKVQTAVWNAGMGVGVGNLTIFPHQWINLRTNNSATIVMPYINSVPMDNMYRHNNLTLMIIPFVPL
17 Q9YLG5|CoxB2    50.9%      TREGPTI---SKGATDVQTAVCNAGMGVGVGNLTIFPHQWINLRTNNSATIVMPYINSVPMDSMIRHNNFTLMIIPFVPL
18 P03313|CoxB3    53.8%      ADKP-VA---SGSNKLVQRVVYNAGMGVGVGNLTIFPHQWINLRTNNSATIVMPYTNSVPMDNMFRHNNVTLMVIPFVPL
19 Q86887|CoxB4    54.2%      EQNA------VTGETAVQTAVCNAGMGVGVGNLTIYPHQWINLRTNNSATIVMPYINSVPMDNMFRHNNFTLMIIPFAPL
20 Q03053|CoxB5    53.8%      DSQN------TTGQTAVQANVINAGMGVGVGNLTIFPHQWINLRTNNSATIVMPYINSVPMDNMFRHNNFTLMIIPFAPL
21 Q9QL88|CoxB6    53.4%      TVEP------ANGQNQVQTAVHNAAMGVAVGNLTIFPHQWINLRTNNSATIVMPYINSVPMDNMFRHNNFTLMIIPFAKL
22 P23008|HRV1A    50.9%      SQERDA------SLRQPSDDSWLNFDGTLLGNLLIFPHQFINLRSNNSATLIVPYVNAVPMDSMLRHNNWSLVIIPISPL
23 P12916|HRV1B    50.9%      GQQRDA------NLRQPSDDSWLNFDGTLLGNLLIFPHQFINLRSNNSATLIVPYVNAVPMDSMLRHNNWSLVIIPISPL
24 P04936|HRV2     49.6%      KAETRLN-----PDLQPTEEYWLNFDGTLLGNITIFPHQFINLRSNNSATIIAPYVNAVPMDSMRSHNNWSLVIIPICPL
25 Q82081|HRV3     49.2%      DTVEV--------AGGPTSDAIYNMDGTLLGNLLIFPHQFINLRTNNTATIVVPYINSVPIDSMTRHNNVSLMVVPIAPL
26 P03303|HRV14    50.0%      SSANE--------VGGPVKDVIYNMNGTLLGNLLIFPHQFINLRTNNTATIVIPYINSVPIDSMTRHNNVSLMVIPIAPL
27 Q82122|HRV16    51.1%      GTQVE-------NEKQPSDDNWLNFDGTLLGNLLIFPHQFINLRSNNSATLIVPYVNAVPMDSMVRHNNWSLVIIPVCQL
28 P03301|Polio1   49.5%      TGTFTPDDNQTSPARRFCPVDYLFGNGTLLGNAFVFPHQIINLRTNNCATLVLPYVNSLSIDSMVKHNNWGIAILPLAPL
29 P06210|Polio2   48.7%      KGSFTLDTNATNPARNFCPVDYLFGSGVLAGNAFVYPHQIINLRTNNCATLVLPYVNSLSIDSMTKHNNWGIAILPLAPL
30 P03302|Polio3   50.2%      YSQFNKDNAVTSPKREFCPVDYLLGCGVLLGNAFVYPHQIINLRTNNSATIVLPYVNALAIDSMVKHNNWGIAILPLSPL
   consensus/100%             ..........................Gh.huphhlhPHQhINLRpNNsATllhPahNshshD..hpH.phslhll.hs.L
   consensus/90%              t.....................h....Gs.lushhlaPHQhINLRoNNsATllhPYhNslshDshh+H.NhslhllPhs.L
   consensus/80%              ttt...........tt.tt.sh.hs.GshlGNhhlaPHQaINLRTNNsATIVhPYlNulPhDsMh+HpNasLhllPhsPL
   consensus/70%              stp..........tttspsssh.ss.GlhlGNLhIaPHQaINLRTNNoATIVhPYlNoVPMDsMhRHsNaoLhlIPhsPL
```

```
                                          β
                        pid 241        .         .         .      ] 276
 1 P32537|EV70    100.0%      AGGTGNTNTYVPITISIAPMCAEYNGLRNAITQ---
 2 Q68T42|EV68     80.8%      GT--RTMSSVVPITVSIAPMCCEFNGLRHAITQ---
 3 B9VUU3|EV71     53.8%      DYDQG-ATPVIPITITLAPMCSEFAGLRQAVTQ---
 4 O91734|Echo1    53.1%      DFSAG-ASTYVPITVTVAPMCAEYNGLRLAGHQ---
 5 Q9YLJ1|Echo5    51.9%      AS-QG-GSTYVPITITIAPMCAEYNGLRLSTQPQ--
 6 Q66474|Echo6    51.9%      DYAAG-SSTYIPITVTVAPMCAEYNGLRLAGHQ---
 7 Q66577|Echo9    51.3%      KS-SG-GTNYVSITITVAPMDAEYNGLRLAGHQ---
 8 P29813|Echo11   51.3%      DYSSD-SSTYVPITVTVAPMCAEYNGLRLSTSLQ--
 9 Q66575|Echo12   51.9%      DFTAE-ASTYVPITVTVAPMCAEYNGLRLASHQ---
10 Q66790|Echo16   51.5%      TS-MG-GSTYVPITVTIAPMCAEYNGLRLSTQHQ--
11 Q9WN78|Echo30   53.1%      EHSPQ-ASTYVPITVTVAPMCAEYNGLRLAGHQ---
12 P21404|CoxA9    53.1%      DYADT-ASTYVPITVTVAPMCAEYNGLRLAQAQ---
13 Q65900|CoxA16   54.2%      DFNAG-ATSEIPITVTIAPMCAEFAGLRQAIKQ---
14 P22055|CoxA21   50.5%      AFAAT-SSPQVPITVTIAPMCTEFNGLRNITIPVHQ
15 P36290|CoxA24   52.2%      DYAPN-SSTEIPITVTIAPMFTEFNGLRNITVPATQ
16 P08291|CoxB1    54.9%      NYSEG-SSPYVPITVTIAPMCAEYNGLRLASSQ---
17 Q9YLG5|CoxB2    50.9%      DYVNG-SSPYIPITVTVAPMSAEYNGLRLASTQ---
18 P03313|CoxB3    53.8%      DYCPG-STTYVPITVTIAPMCAEYNGLRLAGHQ---
19 Q86887|CoxB4    54.2%      DYVTG-ASSYIPITVTVAPMSAEYNGLRLAGHQ---
20 Q03053|CoxB5    53.8%      SYSTG-ATTYVPITVTVAPMCAEYNGLRLAGKQ---
21 Q9QL88|CoxB6    53.4%      AYSDG-ASTFVPITVTIAPMNAEYNGLRLAGHQ---
22 P23008|HRV1A    50.9%      RSETT-SSNIVPITVSISPMCAEFSGARAKNIKQ--
23 P12916|HRV1B    50.9%      RSETT-SSNIRPITVSISPMCAEFSGARAKNVRQ--
24 P04936|HRV2     49.6%      ETSS--AINTIPITISISPMCAEFSGARAKRQ----
25 Q82081|HRV3     49.2%      NAPTG-SSPTLPVTVTIAPMCTEFTGIRSRSIVPQ-
26 P03303|HRV14    50.0%      TVPTG-ATPSLPITVTIAPMCTEFSGIRSKSIVPQ-
27 Q82122|HRV16    51.1%      QSNN--ISNIVPITVSISPMCAEFSGARAKTVVQ--
28 P03301|Polio1   49.5%      NFASE-SSPEIPITLTIAPMCCEFNGLRNITLPRLQ
29 P06210|Polio2   48.7%      DFATE-SSTEIPITLTIAPMCCEFNGLRNITVPRTQ
30 P03302|Polio3   50.2%      DFAQD-SSVEIPITVTIAPMCSEFNGLRNVTAPKFQ
   consensus/100%             t.......s.hslTloluPM.sEasGhR........
   consensus/90%              t...t.uos.lPITloluPMssEasGhR.h......
   consensus/80%              shsst.uos.lPITlTlAPMCsEasGLR.tsh....
   consensus/70%              shsss.uSshlPITVTlAPMCAEaNGLR.tst....
```

Fig. S8. Multiple sequence alignment of capsid proteins of selected enteroviruses. VP1, VP2, VP3 and VP4 protein sequences were aligned using the Clustal Omega tool (4) and viewed using mView (5). Secondary structure elements and structural features according to position in EV70 are shown by color bars over the sequences. Color coding of the elements: yellow - alpha helices; green - beta sheets; black - loops. *(Continued on next page....)*

VP3 1/2

```
                        pid  1                                                                                 80
 1 P32537|EV70      100.0%   GVPTCLLPGSNQFLTTDDHSSAPAFPDFSPTPEMHIPGQVHSMLEIVQIESMMEINNVN--DASGVERLRVQISAQSD-M
 2 Q68T42|EV68       79.1%   GVPTYLLPGSGQFLTTDDHSSAPVLPCFNPTPEMHIPGQIRNMLEMIQVESMMEINNTD--GANGMERLRVDISVQAD-L
 3 B9VUU3|EV71       51.2%   GFPTELKPGTNQFLTTDDGVSAPILPNFHPTPCIHIPGEVRNLLELCQVETILEVNNVPTNATSLMERLRFPVSAQAG-K
 4 O91734|Echo1      47.1%   GLPTMNTPGSNQFLTSDDFQSPSAMPQFDVTPEMHIPGEVRNLMEIAEVDSVMPINNDSAAKVSSMEAYRVELSTNTN-A
 5 Q9YLJ1|Echo5      42.7%   GLPVMNTPGSNQFLTSDDFQSPCAMPEFDVTPPMDIPGEVRNIMEIAEVDSVVPVNNM-SSKVKTIEAYQIPVSVGTTVR
 6 Q66474|Echo6      42.1%   GLPVMSTPGSNQFLTSDDYQSPTAMPQFDVTPEMHIPGEVKNLMEIAEVDSVVPVNNV-NENVNSLEAYRIPVHSVTE-T
 7 Q66577|Echo9      43.2%   GLPTMNTPGSTQFLTSDDFQSPCAMPEFDVTPCMDIPGEVHNLMEIAEVDSVVPVNNT-STHMEGTDAFQIKVTAGNVQD
 8 P29813|Echo11     42.5%   GLPVMNTPGSNQFLTSDDFQSPSAMPQFDVTPELNIPGEVQNLMEIAEVDSVVPVNNV-EGKLDTMEVYRIPVQSGNH-Q
 9 Q66575|Echo12     42.3%   GLPTMNTPGSNQFLTSDDFQSPSAMPQFDVTPELRIPGEVKNLMEIAEVDSVVPVNNT-QDSVYNMDVYKIPVSGGNQ-L
10 Q66790|Echo16     42.0%   GLPVMNVPGSNQFLTSDNFQSPCAMPEYDVTPPLDIPGEVNNLMEVAEVDSVVPVNNL-SDNVKTIKAYQIPVSAGDSSR
11 Q9WN78|Echo30     42.9%   GLPTMNTPGSTQFLTSDDFQSPSAMPQFDVTPEIQIPGQVRNLMEIAEVDSVVPVDNT-EEHVNSIEAYRIPVRPQTN-S
12 P21404|CoxA9      42.9%   GLPTMNTPGSTQFLTSDDFQSPCALPQFDVTPSMNIPGEVKNLMEIAEVDSVVPVNNV-QDTTDQMEMFRIPVTINAP-L
13 Q65900|CoxA16     50.4%   GIPTELKPGTNQFLTTDDGVSAPILPGFHPTPAIHIPGEVRNLLEICRVETILEVNNLQSNETTPMQRLCFPVSVQSK-T
14 P22055|CoxA21     41.2%   GLPTMNTPGSNQFLTSDDFQSPCALPNFDVTPPIHIPGEVKNMMELAEIDTLIPMNAV-DGKVNTMEMYQIPLNDNL--S
15 P36290|CoxA24     43.3%   GLPTMLTPGSSQFLTSDDFQSPCALPNFDVTPPIHIPGEVFNMMELAEIDSMIPMNSV-TGKANTMEMYPIPLDDKG--G
16 P08291|CoxB1      44.2%   GLPVMTTPGSTQFLTSDDFQSPSAMPQFDVTPEMQIPGRVNNLMEIAEVDSVVPVNNT-DNNVNGLKAYQIPVQSNSD-N
17 Q9YLG5|CoxB2      48.3%   GLPTMLTPGSNQFLTSDDFQSPSAMPQFDVTPEMNIPGRVHNLMEIAEVDSVVPLNNI-QDNLRKMDIYRVQVSSQTS-Q
18 P03313|CoxB3      44.6%   GLPTMNTPGSCQFLTSDDFQSPSAMPQYDVTPEMRIPGEVKNLMEIAEVDSVVPVQNV-GEKVNSMEAYQIPVRSNEG-S
19 Q86887|CoxB4      46.2%   GLPTMLTPGSTQFLTSDDFQSPSAMPQFDVTPEMNIPGQVRNLMEIAEVDSVVPINNL-QANLKTMEAYRVQVRSTDE-M
20 Q03053|CoxB5      46.7%   GLPTMLTPGSNQFLTSDDFQSPSAMPQFDVTPEMAIPGQVNNLMEIAEVDSVVPVNNT-EGKVSSIEAYQIPVQSNST-N
21 Q9QL88|CoxB6      45.0%   GLPVMTTPGSTQFLTSDDFQSPCAMPQFDVTPEMNIPGQVNNLMEIAEVDSVVPVNNT-ETNVNGMDAYRIPVQSNMD-T
22 P23008|HRV1A      45.0%   GLPVYITPGSGQFMTTDDMQSPCALPWYHPTKEISIPGEVKNLIEMCQVDTLIPVNNV-GNNVGNVSMYTVQLGNQTG-M
23 P12916|HRV1B      45.0%   GLPVYITPGSGQFMTTDDMQSPCALPWYHPTKEISIPGEVKNLIEMCQVDTLIPVNNV-GTNVGNISMYTVQLGNQMD-M
24 P04936|HRV2       43.2%   GLPVFITPGSGQFLTTDDFQSPCALPWYHPTKEISIPGEVKNLVEICQVDSLVPINNT-DTYINSENMYSVVLQSSIN-A
25 Q82081|HRV3       42.3%   GLPTTTLPGSGQFLTTDDRQSPSALPSYEPTPRIHIPGKVRNLLEIIQVGTLIPMNNT-GTNDNVTN-YLIPLH-ADR-Q
26 P03303|HRV14      42.7%   GLPTTTLPGSGQFLTTDDRQSPSALPNYEPTPRIHIPGKVHNLLEIIQVDTLIPMNNT-HTKDEVNS-YLIPLN-ANR-Q
27 Q82122|HRV16      41.7%   GLPVYVTPGSGQFMTTDDMQSPCALPWYHPTKEIFIPGEVKNLIEMCQVDTLIPINST-QSNIGNVSMYTVTLSPQTK-L
28 P03301|Polio1     42.1%   GLPVMNTPGSNQYLTADNFQSPCALPEFDVTPPIDIPGEVKNMMELAEIDTMIPFDLS-AKKKNTMEMYRVRLSDKPH-T
29 P06210|Polio2     41.3%   GLPVLNTPGSNQYLTADNYQSPCAIPEFDVTPPIDIPGEVRNMMELAEIDTMIPLNLT-NQRKNTMDMYRVELNDAAH-S
30 P03302|Polio3     42.1%   GLPVLNTPGSNQYLTSDNHQSPCAIPEFDVTPPIDIPGEVKNMMELAEIDTMIPLNLE-STKRNTMDMYRVTLSDSAD-L
   consensus/100%            GhPs..hPGosQahTsDsh.SsshhP.apsT..h.IPGpl.shhEhhpltohh.hp..........p.h.h.lt......
   consensus/90%             GlPsh.hPGSsQFLToDshpSssAhP.acsT..hpIPGpVpNhhEhspl-ohl.hNsh.ttphtshphhpl.lpst....
   consensus/80%             GLPsh.sPGSsQFLToDDhQSPsAhPpacsTP.hpIPGcV+NhhElspVDollPlNNs.ttphpshphYpl.lpstst..
   consensus/70%             GLPsh.TPGSsQFLToDDaQSPsAhPpF-sTPphpIPGcV+NLhElscVDollPlNNs.pspsssh-hYplslpspsp.t

                        pid  81                                                                                16
 1 P32537|EV70      100.0%   DQLLFNIPLDIQLEGPLRNTLLGNISRYYTHWSGSLEMTFMFCGSFMTTGKLIICYTPPGGSSPTDRMQAMLATHVVWDF
 2 Q68T42|EV68       79.1%   DQLLFNIPLDIQLDGPLRNTLVGNISRYYTHWSGSLEMTFMFCGSFMATGKLILCYTPPGGSCPTTRETAMLGTHIVWDF
 3 B9VUU3|EV71       51.2%   GELCAVFRADPGRNGPWQSTLLGQLCGYYTQWSGSLEVTFMFTGSFMATGKMLIAYTPPGGPLPKDRATAMLGTHVIWDF
 4 O91734|Echo1      47.1%   GTQVFGFQLNPGAESVMNRTLMGEILNYYAHWSGSIKITFVFCGSAMTTGKFLLSYAPPGAGAPKTRKDAMLGTHVVWDV
 5 Q9YLJ1|Echo5      42.7%   GDAIFSFQLNPGNSPVLNRTLLGEIINYYAHWSGSIKLTFLFCGSAMATGKLLLAYSPPGASVPTSRKDAMLGTHIIWDL
 6 Q66474|Echo6      42.1%   GAQVFGFTLQPGADSVMERTLHGEILNYYANWSGSIKLTFMYCGSAMATGKFLLAYSPPGAGVPKNRKEAMLGTHMIWDI
 7 Q66577|Echo9      43.2%   KSAIFSFQLNPGNSTVLRRTLLGEILNYYAHWSGSIKLTFLFCGSAMATGKLLLAYSHAGASVPKSRRDAMLGTHVIWDV
 8 P29813|Echo11     42.5%   SDQVFGFQVQPGLDSVFKHTLLGEILNYFAHWSGSIKLTFVFCGSAMATGKFLLAYAPPGANAPKNRKDAMLGTHIIWDV
 9 Q66575|Echo12     42.3%   STQVFGFQMQPGLNSVFKRTLLGEILNYYAHWSGSVKLTFVFCGSAMALAKFLLAYSPPGADPPKSRKEAMLGTHVIWDI
10 Q66790|Echo16     42.0%   PEAVFKFQLDPGSGSVLKHTLLGEIINYYAHWSGSIKLTFVFCGSAMATGKLLIAYSPPGASAPATRKDAMLGTHIIWDL
11 Q9WN78|Echo30     42.9%   GEQVFGFQLQPGYDSVLKHTLLGEILNYYANWSGSMKLTFMYCGAAMATGKFLIAYSPPGAGVPGSRKDAMLGTHVIWDV
12 P21404|CoxA9      42.9%   QQQVFGLRLQPGLDSVFKHTLLGEILNYYAHWSGSMKLTFVFCGSAMATGKFLIAYSPPGANPPKTRKDAMLGTHIIWDI
13 Q65900|CoxA16     50.4%   GELCAVFRADPGRNGPWQSTILGQLCRYYTQWSGSLEVTFMFAGSFMATGKMLIAYTPPGGGVPADRLTAMLGTHVIWDF
14 P22055|CoxA21     41.2%   KAPIFCLSLSPASDKRLSHTMLGEILNYYTHWTGSIRFTFLFCGSMMATGKLLLSYSPPGAKPPTNRKDAMLGTHIIWDL
15 P36290|CoxA24     43.3%   KTPIFSISLSPASDKRLQYTMLGEILNYYTHWTGSLRFTFLFCGSMMATGKILLSYSPPGAKPPTTRKDAMLGTHIIWDL
16 P08291|CoxB1      44.2%   RRQVFGFPLQPGANNVLNRTLLGEILNYYTHWSGSIKLTFMFCGSAMATGKFLLAYSPPGAGVPKNRRDAMLGTHVIWDV
17 Q9YLG5|CoxB2      48.3%   GAQVFGFSLQPGASSVLQRTLLGEILNYYTHWSGSLKLTFVFCGSAMATGKFLLAYSPPGAGVPPDRKKAMLGTHVIWDV
18 P03313|CoxB3      44.6%   GTQVFGFPLQPGYSSVFSRTLLGEILNYYTHWSGSIKLTFMFCGSAMATGKFLLAYSPPGAGAPTKRVDAMLGTHVIWDV
19 Q86887|CoxB4      46.2%   GGQIFGFPLQPGASSVLQRTLLGEILNYYTHWSGSLKLTFVFCGSAMATGKFLLAYSPPGAGAPDSRKNAMLGTHVIWDV
20 Q03053|CoxB5      46.7%   GSQVFGFPLIPGASSVLNRTLLGEILNYYTHWSGSIKLTFMFCGSAMATGKFLLAYSPPGAGAPTTRKEAMLGTHVIWDV
21 Q9QL88|CoxB6      45.0%   GGQVFGFPLQPGASSVFQRTLLGEILNYYTHWSGSIKLTFMFCGSAMATGKFLLAYSPPGAGAPKSRKDAMLGTHVIWDV
22 P23008|HRV1A      45.0%   AQKVFSIKVDITS-QPLATTLIGEIASYYTHWTGSLRFSFMFCGTANTTLKLLLAYTPPGIDEPTTRKDAMLGTHVVWDV
23 P12916|HRV1B      45.0%   AQEVFAIKVDITS-QPLATTLIGEIASYYTHWTGSLRFSFMFCGTANTTLKLLLAYTPPGIDKPATRKDAMLGTHVVWDV
24 P04936|HRV2       43.2%   PDKIFSIRTDVAS-QPLATTLIGEISSYFTHWTGSLRFSFMFCGTANTTVKLLLAYTPPGIAEPTTRKDAMLGTHVIWDV
25 Q82081|HRV3       42.3%   NEQIFGTKLYIGD-GVFKTTLLGEIAQYYTHWSGSLRISLMYTGPALSSAKIILAYTPPGTRGPEDRKEAMLGTHVVWDI
26 P03303|HRV14      42.7%   NEQVFGTNLFIGD-GVFKTTLLGEIVQYYTHWSGSLRFSLMYTGPALSSAKLILAYTPPGARGPQDRREAMLGTHVVWDI
27 Q82122|HRV16      41.7%   AEEIFAIKVDIAS-HPLATTLIGEIASYFTHWTGSLRFSFMFCGTANTTLKVLLAYTPPGIGKPRSRKEAMLGTHVVWDV
28 P03301|Polio1     42.1%   DDPILCLSLSPASDPRLSHTMLGEILNYYTHWAGSLKFTFLFCGSMMATGKLLVSYAPPGADPPKKRKEAMLGTHVIWDI
29 P06210|Polio2     41.3%   DTPILCLSLSPASDPRLAHTMLGEILNYYTHWAGSLKFTFLFCGSMMATGKLLVSYAPPGAEAPKSRKEAMLGTHVIWDI
30 P03302|Polio3     42.1%   SQPILCLSLSPASDPRLSHTMLGEVLNYYTHWAGSLKFTFLFCGSMMATGKILVAYAPPGAQPPTSRKEAMLGTHVIWDL
   consensus/100%            .t.hh.h.h..t....ht.ThhGpl.tYaspWsGShchohhasGsh.s.hKhllsYs.sGh..P.pR.pAMLuTHhlWDh
   consensus/90%             tt.lhsh.hp.s....htpThlGpIhpYYspWoGSlchoFhasGoh.sTsKhlluYsPPGht.PtsRtpAMLGTHllWDh
   consensus/80%             sp.lFsh.lp.u.tsshppTLlGEIhsYYsHWoGSl+hTFhFCGohMuTuKhLluYoPPGussPpsR+-AMLGTHllWDl
   consensus/70%             sptlFuhpLpPussssshppTLLGEIlsYYTHWSGSl+hTFhFCGSAMATGKhLLAYoPPGAssPpsRK-AMLGTHVIWDl
```

Fig. S8. Multiple sequence alignment of capsid proteins of selected enteroviruses. VP1, VP2, VP3 and VP4 protein sequences were aligned using the Clustal Omega tool (4) and viewed using mView (5). Secondary structure elements and structural features according to position in EV70 are shown by color bars over the sequences. Color coding of the elements: yellow - alpha helices; green - beta sheets; black - loops. *(Continued on next page....)*

VP3 2/2

```
                                 β                       β                β
                pid  161                                                                                  240
 1 P32537|EV70     100.0%   GLQSSITIIIPWISGSHYRMFNTDAKAINANVGYVTCFMQTNLVAPVGAADQCYIVGMVAAKKDFNLRLMRDSPDIGQSA
 2 Q68T42|EV68      79.1%   GLQSSITLIIPWISGSHYRMFNSDAKSTNANVGYVTCFMQTNLIVPSESSDTCSLIGFIAAKDDFSLRLMRDSPDIGQ--
 3 B9VUU3|EV71      51.2%   GLQSSVTLVIPWISNTHYRAHARDGVFDYYTTGLVSIWYQTNYVVPIGAPNTAYIIALAAAQKNFTMKLCKDASDILQTG
 4 O91734|Echo1     47.1%   GLQSSCVLCIPWISQTHYRFVEKDP---YTNAGFVTCWYQTSVVSPASNQPKCYMMCMVSACNDFSVRMLRDTKFIEQTS
 5 Q9YLJ1|Echo5     42.7%   GLQSSCVLCVPWISQTHYRMVQQDE---YSAAGYITCWYQTNIIVPPDTPTDCIVLCFVSACNDFSVRMLKDTPFVEQEA
 6 Q66474|Echo6     42.1%   GLQSRCVLCVPWISQTHYRFVSKDS---YTDAGFITCWYQTSIVVPAEVQNQSVILCFVSACNDFSVRLLRDSPFVTQTA
 7 Q66577|Echo9     43.2%   GLQSSCVLCVPWISQTHYRLVAQDE---YTSAGYITCWYQTNIVVPPETPSDCVVLCFVSACNDFSVRMLKDTPFIEQAA
 8 P29813|Echo11    42.5%   GLQSSCVLCVPWISQTHYRLVQQDE---YTSAGNVTCWYQTGIVVPAGTPTSCSIMCFVSACNDFSVRLLKDTPFIEQTA
 9 Q66575|Echo12    42.3%   GLQSSCVLCVPWISQTHYRLVQQDE---YTSAGYVTCWYQTSLVVPPGAPATCGVLCLASACNDFSVRMLRDTPFIEQKQ
10 Q66790|Echo16    42.0%   GLQSSCVLCVPWISQTHYRMVERDE---YTTAGYISCWYQTNIIVPPDTPSQCYMLCLASACNDFSVRMLKDTPFIQQEA
11 Q9WN78|Echo30    42.9%   GLQSSCVLCVPWISQTNYRYVTRDA---YTDAGYITCWYQTSIVTPPDIPTTSTILCFVSACNDFSVRLLRDTPFITQQA
12 P21404|CoxA9     42.9%   GLQSSCVLCVPWISQTHYRLVQQDE---YTSAGYVTCWYQTGMIVPPGTPNSSSIMCFASACNDFSVRMLRDTPFISQDN
13 Q65900|CoxA16    50.4%   GLQSSVTLVIPWISNTHYRAHAKDGYFDYYTTGTITIWYQTNYVVPIGAPTTAYIVALAAAQDNFTMKLCKDTEDIEQSA
14 P22055|CoxA21    41.2%   GLQSSCSMVAPWISNTVYRRCARDD---FTEGGFITCFYQTRIVVPASTPTSMFMLGFVSACPDFSVRLLRDTSHISQSK
15 P36290|CoxA24    43.3%   GLQSSCTMLAPWISNTVYRRCIKDD---FTEGGYITCFYQTRIVVPSGTPTSMFMLAFVSACPDFSVRLLRDTNHISQRT
16 P08291|CoxB1     44.2%   GLQSSCVLCVPWISQTHYRYVVEDE---YTAAGYVTCWYQTNIIVPADVQSTCDILCFVSACNDFSVRMLKDTPFIRQDN
17 Q9YLG5|CoxB2     48.3%   GLQSSCVLCVPWISQTHYRYTVKDE---YTDSGYITCWYQTNVIAPADALSTCYIMCMVSACNDFSVRMLRDTRFIKQTA
18 P03313|CoxB3     44.6%   GLQSSCVLCIPWISQTHYRFVASDE---YTAGGFITCWYQTNIVVPADAQSSCYIMCFVSACNDFSVRLLKDTPFISQQN
19 Q86887|CoxB4     46.2%   GLQSSCVLCVPWISQTHYRYVVDDK---YTASGFISCWYQTNVIVPAEAQKSCYIMCFVSACNDFSVRMLRDTQFIKQDT
20 Q03053|CoxB5     46.7%   GLQSSCVLCIPWISQTHYRYVVVDE---YTAGGYITCWYQTNIVVPADTQSDCKILCFVSACNDFSVRMLKDTPFIKQDS
21 Q9QL88|CoxB6     45.0%   GLQSSCVLCIPWISQTHYRFVVADE---YTAGGFITCWYQTNVIVPLGAQSNCSILCFVSACNDFSVRMLRDTKFISQTA
22 P23008|HRV1A     45.0%   GLQSTISLVVPWVSASHFRLTADNK---YSMAGYITCWYQTNLVVPPSTPQTADMLCFVSACKDFCLRMARDTDLHIQSG
23 P12916|HRV1B     45.0%   GLQSTISLVVPWVSASHFRLTANDK---YSMAGYITCWYQTNLVVPPNTPQTADMLCFVSACKDFCLRMARDTDLHIQSG
24 P04936|HRV2      43.2%   GLQSTISMVVPWISASHYRNTSPGR----STSGYITCWYQTRLVIPPQTPPTARLLCFVSGCKDFCLRMARDTNLHLQSG
25 Q82081|HRV3      42.3%   GLQSTIVMTIPWTSGVQFRYTDPDT---YTSAGYLSCWYQTSLILPPQTSGQVYLLSFISACPDFKLRLMKDTQTISQTD
26 P03303|HRV14     42.7%   GLQSTIVMTIPWTSGVQFRYTDPDT---YTSAGFLSCWYQTSLILPPETTGQVYLLSFISACPDFKLRLMKDTQTISQTV
27 Q82122|HRV16     41.7%   GLQSTVSLVVPWISASQYRFTTPDT---YSSAGYITCWYQTNFVVPPNTPNTAEMLCFVSGCKDFCLRMARDTDLHKQTG
28 P03301|Polio1    42.1%   GLQSSCTMVVPWISNTTYRQTIDDS---FTEGGYISVFYQTRIVVPLSTPREMDILGFVSACNDFSVRLMRDTTHIEQKA
29 P06210|Polio2    41.3%   GLQSSCTMVVPWISNTTYRQTINDS---FTEGGYISMFYQTRVVVPLSTPRKMDILGFVSACNDFSVRLLRDTTHISQEA
30 P03302|Polio3    42.1%   GLQSSCTMVVPWISNVTYRQTTQDS---FTEGGYISMFYQTRIVVPLSTPKSMSMLGFVSACNDFSVRLLRDTTHISQSA
   consensus/100%           GLQSphshhhPWhSts.aR.h..s.......sG.lohahQTthl.P.t...p..hhshhuup.sFph+hh+Ds..h.Q..
   consensus/90%            GLQSohshslPWlStopaR.h..Dt...astsGalohaYQTphlsP.ts.tph.hhshsuAp.DFslRhh+Do..h.Qps
   consensus/80%            GLQSohshslPWIStopYRhs.pDp...aotuGaloCaYQTslllPsts.sps.hlshlSACsDFslRhh+DT.hIpQps
   consensus/70%            GLQSSssLslPWISpTHYRhsspDp...YTsuGalTCWYQTsllVPsssssss.llsFVSACsDFSlRhh+DTshIpQss

                pid  241       ] 246
 1 P32537|EV70     100.0%   IL----
 2 Q68T42|EV68      79.1%   ------
 3 B9VUU3|EV71      51.2%   TIQGD-
 4 O91734|Echo1     47.1%   F-----
 5 Q9YLJ1|Echo5     42.7%   D-----
 6 Q66474|Echo6     42.1%   F-----
 7 Q66577|Echo9     43.2%   E-----
 8 P29813|Echo11    42.5%   L-----
 9 Q66575|Echo12    42.3%   ------
10 Q66790|Echo16    42.0%   KLQ---
11 Q9WN78|Echo30    42.9%   L-----
12 P21404|CoxA9     42.9%   K-----
13 Q65900|CoxA16    50.4%   NIQGD-
14 P22055|CoxA21    41.2%   LIARTQ
15 P36290|CoxA24    43.3%   LFARAQ
16 P08291|CoxB1     44.2%   F-----
17 Q9YLG5|CoxB2     48.3%   F-----
18 P03313|CoxB3     44.6%   F-----
19 Q86887|CoxB4     46.2%   F-----
20 Q03053|CoxB5     46.7%   F-----
21 Q9QL88|CoxB6     45.0%   F-----
22 P23008|HRV1A     45.0%   P-----
23 P12916|HRV1B     45.0%   P-----
24 P04936|HRV2      43.2%   AI----
25 Q82081|HRV3      42.3%   ------
26 P03303|HRV14     42.7%   ------
27 Q82122|HRV16     41.7%   P-----
28 P03301|Polio1    42.1%   LAQ---
29 P06210|Polio2    41.3%   MPQ---
30 P03302|Polio3    42.1%   LPQ---
   consensus/100%           ......
   consensus/90%            ......
   consensus/80%            ......
   consensus/70%            ......
```

Fig. S8. Multiple sequence alignment of capsid proteins of selected enteroviruses. VP1, VP2, VP3 and VP4 protein sequences were aligned using the Clustal Omega tool (4) and viewed using mView (5). Secondary structure elements and structural features according to position in EV70 are shown by color bars over the sequences. Color coding of the elements: yellow - alpha helices; green - beta sheets; black - loops. *(Continued on next page....)*

VP4

```
                                                                α                α
                      pid   1 [         .         .         .         .         .         .        ] 69
 1 P32537|EV70     100.0%    -GAQVSRQQTGTHENANVATGGSSITYNQINFYKDSYAASASKQDFSQDPSKFTEPVAEALKAGAPVLK
 2 Q68T42|EV68      89.9%    MGAQVTRQQTGTHENANIATNGSHITYNQINFYKDSYAASASKQDFSQDPSKFTEPVVEGLKAGAPVLK
 3 B9VUU3|EV71      55.9%    -GSQVSTQRSGSHENSNSATEGSTINYTTINYYKDSYAATAGKQSLKQDPDKFANPVKDIFTEMAAPLK
 4 O91734|Echo1     52.9%    -GAQVSTQKTGAHETSLSATGNSIIHYTNINYYKDAASNSANRQDFTQDPGKFTEPMKDVMIKTLPALN
 5 Q9YLJ1|Echo5     52.9%    -GAQVSTQKTGAHETLLEAAHGATINYTNINYYKDAASNSANRQDFSQDPGKFTEPVKDLMIKSMPALN
 6 Q66474|Echo6     52.9%    -GAQVSTQKTGAHETGLSASGNSIIHYTNINYYKDAASNSANRQDFTQDPGKFTEPVKDIMAKTLPALN
 7 Q66577|Echo9     55.9%    -GAQVSTQKTGAHETGLNASGNSIIHYTNINYYKDSASNSANRQDFTQDPSKFTEPVKDVMIKTLPALN
 8 P29813|Echo11    52.9%    -GAQVSTQKTGAHETGLNASGSSIIHYTNINYYKDAASNSANRQEFSQDPGKFTEPVKDIMVKSLPALN
 9 Q66575|Echo12    52.9%    -GAQVSTQKTGAHETGLSASGNSIIHYTNINYYKDAASNSANRQDFTQDPGKFTEPVKDIMIKSMPALN
10 Q66790|Echo16    54.4%    -GAQVSTQKTGAHETLLEAAQGATINYTNINYYKDAASNSANRQDFSQDPSKFTEPVKDIMIKSMPALN
11 Q9WN78|Echo30    54.4%    -GAQVSTQKTGAHETGLSASGNSVIHYTNINYYKDSASNSLNRQDFTQDPSRFTEPVQDVLIKTLPALN
12 P21404|CoxA9     54.4%    -GAQVSTQKTGAHETSLSAAGNSIIHYTNINYYKDAASNSANRQDFTQDPSKFTEPVKDVMIKSLPALN
13 Q65900|CoxA16    58.8%    -GSQVSTQRSGSHENSNSASEGSTINYTTINYYKDAYAASAGRQDMSQDPKKFTDPVMDVIHEMAPPLK
14 P22055|CoxA21    61.8%    -GAQVSTQKTGAHENQNVAANGSTINYTTINYYKDSASNSATRQDLSQDPSKFTEPVKDLMLKTAPALN
15 P36290|CoxA24    63.2%    -GAQVSSQKVGAHENTNVATGGSTVNYTTINYYKDSASNAASKLDFSQDPSKFTEPVKDIMLKSAPALN
16 P08291|CoxB1     52.9%    -GAQVSTQKTGAHETGLNASGNSIIHYTNINYYKDAASNSANRQDFTQDPGKFTEPVKDIMIKSMPALN
17 Q9YLG5|CoxB2     54.4%    -GAQVSTQKTGAHETGLSASGGSIIHYTNINYYKDAASNSANRQDFTQDPGKFTEPVKDIMIKSMPALN
18 P03313|CoxB3     52.9%    -GAQVSTQKTGAHETRLNASGNSIIHYTNINYYKDAASNSANRQDFTQDPGKFTEPVKDIMIKSLPALN
19 Q86887|CoxB4     55.9%    -GAQVSTQKTGAHETSLSATGNSIIHYTNINYYKDAASNSANRQDFTQDPSKFTEPVKDVMIKSLPALN
20 Q03053|CoxB5     51.5%    -GAQVSTQKTGAHETGLRASGNSIIHYTNINYYKDAASNSANRQEFAQDPGKFTEPVKDIMIKSMPALN
21 Q9QL88|CoxB6     55.9%    -GAQVSTQKTGAHETALNAQGNSVIHYTNINYYKDAASNSANRQDFTQDPSKFTEPVKDVMIKSLPALN
22 P23008|HRV1A     54.4%    -GAQVSRQNVGTHSTQNSVSNGSSLNYFNINYFKDAASSGASRLDFSQDPSKFTDPVKDVLEKGIPTLQ
23 P12916|HRV1B     54.4%    -GAQVSRQNVGTHSTQNSVSNGSSLNYFNINYFKDAASSGASRLDFSQDPSKFTDPVKDVLEKGIPTLQ
24 P04936|HRV2      52.9%    -GAQVSRQNVGTHSTQNSVSNGSSLNYFNINYFKDAASNGASKLEFTQDPSKFTDPVKDVLEKGIPTLQ
25 Q82081|HRV3      54.4%    -GAQVSTQKSGSHENQNILTNGSNQTFTVINYYKDAASSSAGQSFSMDPSKFTEPVKDLMLKGAPALN
26 P03303|HRV14     52.9%    -GAQVSTQKSGSHENQNILTNGSNQTFTVINYYKDAASTSSAGQSLSMDPSKFTEPVKDLMLKGAPALN
27 Q82122|HRV16     54.4%    -GAQVSRQNVGTHSTQNMVSNGSSLNYFNINYFKDAASSGASRLDFSQDPSKFTDPVKDVLEKGIPTLQ
28 P03301|Polio1    60.3%    -GAQVSSQKVGAHENSNRAYGGSTINYTTINYYRDSASNAASKQDFSQDPSKFTEPIKDVLIKTSPMLN
29 P06210|Polio2    60.3%    -GAQVSSQKVGAHENSNRAYGGSTINYTTINYYRDSASNAASKQDFAQDPSKFTEPIKDVLIKTAPTLN
30 P03302|Polio3    61.8%    -GAQVSSQKVGAHENSNRAYGGSTINYTTINYYKDSASNAASKQDYSQDPSKFTEPLKDVLIKTAPALN
   consensus/100%            .GuQVopQpsGsHps...h.tsu..pa..INaa+Duhuss.st.pht.DPt+FspPh.-hh.t..s.Lp
   consensus/90%             .GAQVSpQpsGsHpst..sttsS.lpYhsINYaKDuhusuAs+.-hoQDPuKFT-PlpDlh.cshPsLp
   consensus/80%             .GAQVSoQ+sGuHEst..AsssS.lpYTsINYYKDuASsuAs+QDFoQDPuKFTEPVKDlhhKshPsLp
   consensus/70%             .GAQVSoQKoGuHEst.pAossShIpYTsINYYKDuASNSAs+QDFoQDPuKFTEPVKDlhlKshPsLN
```

Fig. S8. Multiple sequence alignment of capsid proteins of selected enteroviruses. VP1, VP2, VP3 and VP4 protein sequences were aligned using the Clustal Omega tool (4) and viewed using mView (5). Secondary structure elements and structural features according to position in EV70 are shown by color bars over the sequences. Color coding of the elements: yellow - alpha helices; green - beta sheets; black - loops.

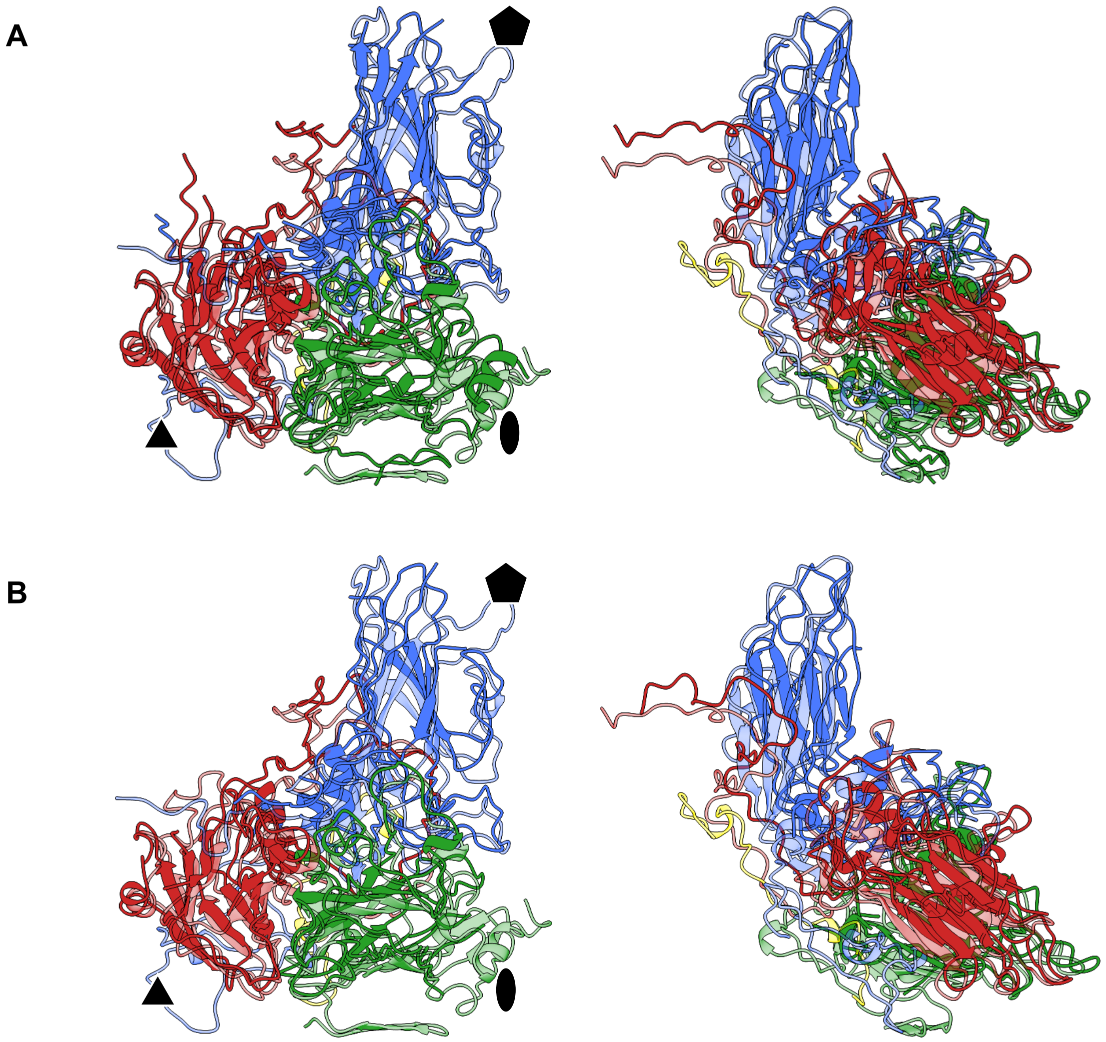

Fig. S9. Comparison of native EV70 virion protomer with altered particle protomer (A) and empty particle protomer (B). Cartoon representations of the protomers are overlaid to show the expansion of altered and empty particles mediated by the movement of the protomers away from the particle center. The native particle protomer is shown in semi-transparent colors. Selected fivefold, threefold and twofold axes of symmetry are indicated with pentagon, triangle and oval respectively.

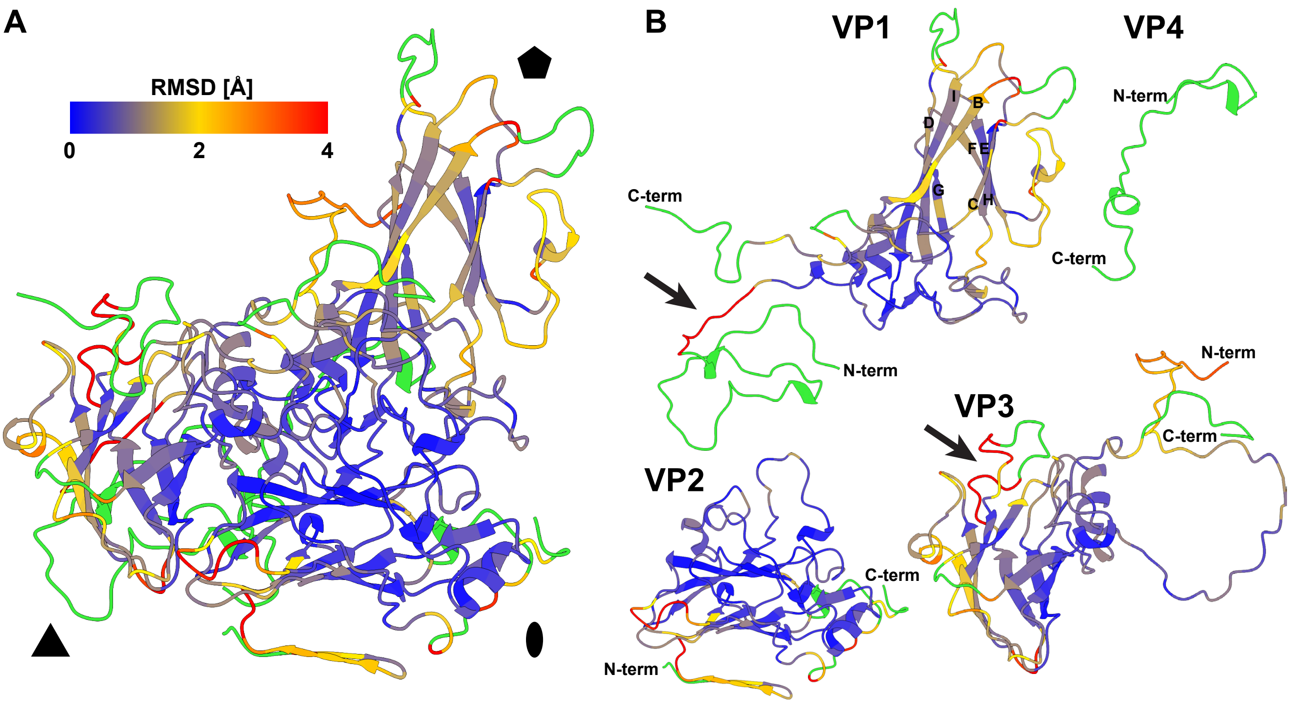

Fig. S10. Root-mean-square deviation (RMSD) of peptide backbone of EV70 virion and activated particle. (A) Protomer of capsid proteins from EV70 virion colored according to RMSD values comparing distances of residues in virion and activated particle. (B) Individual capsid proteins are colored according to RMSD values. Residues that are not resolved in the structure of the altered particle are shown in bright green. Arrows indicate the part of VP1 that is externalized in the A-particle and the part of VP3 that undergoes significant movement to enable this process.

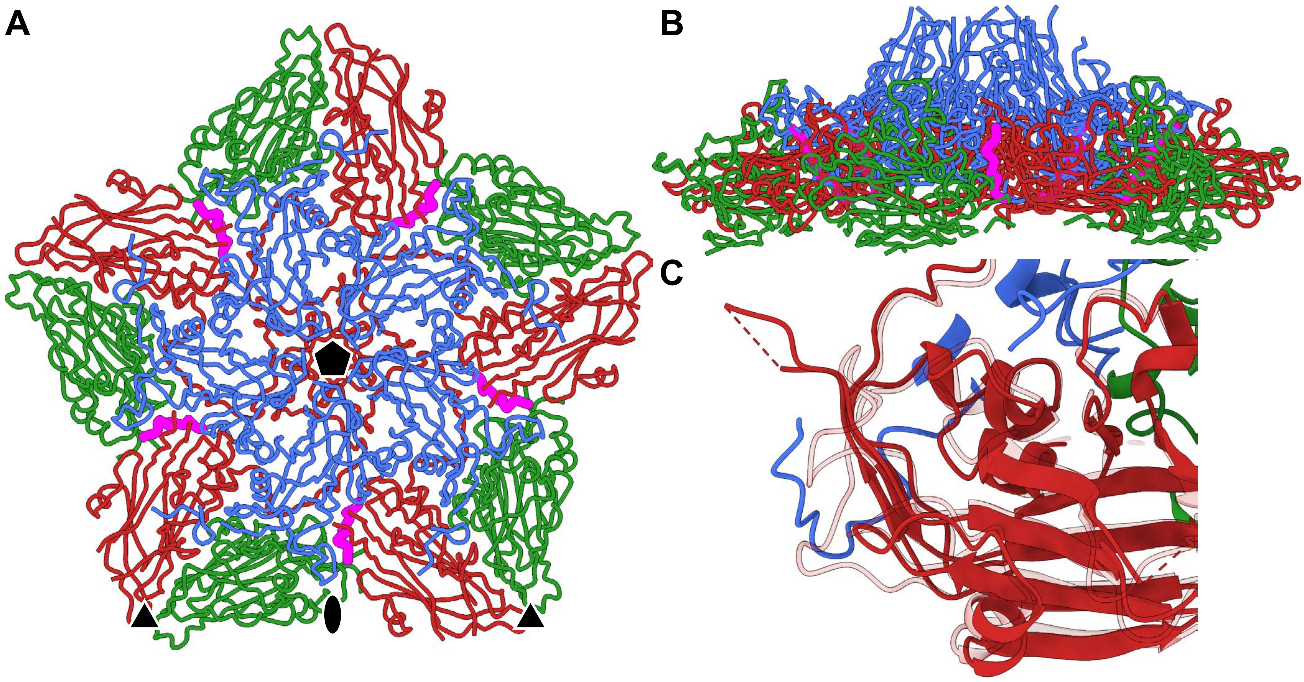

Fig. S11. Externalization of N-terminal arm of VP1 in altered particle of EV70. (AB) Ribbon representation of protomers forming one pentamer in capsid of altered particle viewed from outside particle (A) and rotated 90 degrees about x-axis (B). VP1 is shown in blue, VP2 in green and VP3 in red. The part of the N-terminal arm of VP1 passing through the capsid is highlighted in magenta. Positions of selected fivefold, threefold, and twofold axes of symmetry of the capsid are indicated with a pentagon, triangle, and oval, respectively. The externalization of VP1 N-termini occurs between a twofold axis and the "canyon". (C) Rearrangement of GH loop of VP3 is required to enable externalization of N-terminus of VP1. Cartoon representation of VP1 of the altered particle is shown in blue, VP2 in green, VP3 in red, and the superimposed structure of VP3 from the virion is shown in semi-transparent red.

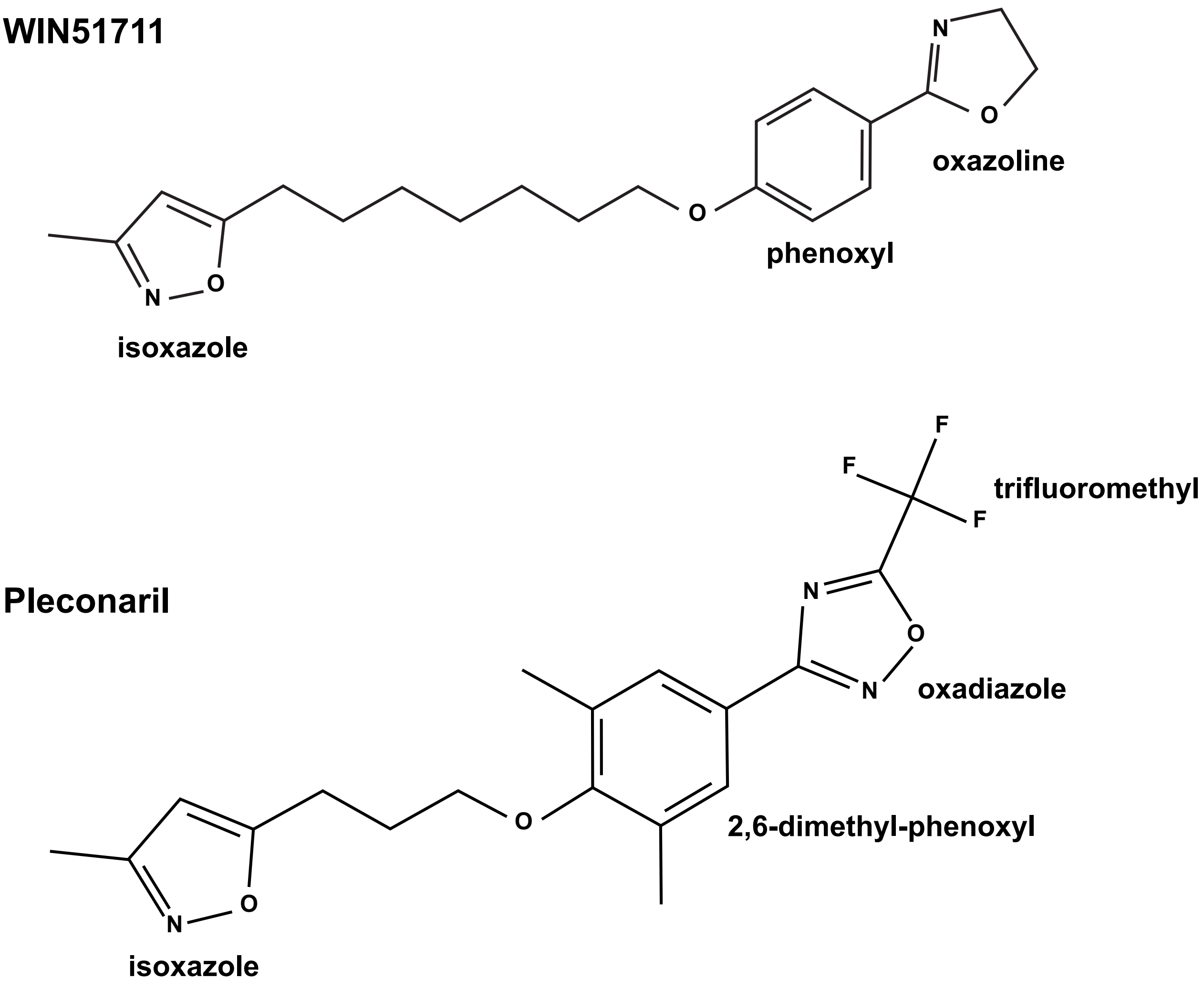

Fig. S12. Schematic drawing of WIN51711 (Disoxaril) and Pleconaril molecular structure.

A

Interfacing residues

Inaccessible residues
Solvent-accessible residues

HSDC Residues making **H**ydrogen/**D**isulphide bond, **S**alt bridge or **C**ovalent link
Interfacing residues

ASA Accessible Surface Area, Å² **BSA** Buried Surface Area, Å² **Δ^iG** Solvation energy effect, kcal/mol |||| Buried area percentage, one bar per 10%

<table>
<tr><th>##</th><th>Structure 1</th><th>HSDC</th><th>ASA</th><th>BSA</th><th>Δ^iG</th></tr>
<tr><td>VP1</td><td>WIN51711:W71 301</td><td></td><td>635.10</td><td>598.89 ||||||||||</td><td>0.30</td></tr>
<tr><td>VP3</td><td>WIN51711:W71 301</td><td></td><td>635.10</td><td>36.21 |</td><td>-0.67</td></tr>
<tr><td>VP3*</td><td>WIN51711:W71 301</td><td></td><td>641.00</td><td>17.12 |</td><td>-0.57</td></tr>
</table>

<table>
<tr><th>##</th><th>Structure 2</th><th>HSDC</th><th>ASA</th><th>BSA</th><th>Δ^iG</th></tr>
<tr><td>1</td><td>VP1:VAL 74</td><td></td><td>4.89</td><td>3.86 ||||||||</td><td>0.06</td></tr>
<tr><td>2</td><td>VP1:TRP 98</td><td></td><td>19.44</td><td>9.37 |||||</td><td>0.15</td></tr>
<tr><td>3</td><td>VP1:LEU 100</td><td></td><td>40.28</td><td>34.76 |||||||||</td><td>0.56</td></tr>
<tr><td>4</td><td>VP1:ASN 101</td><td></td><td>34.79</td><td>7.87 |||</td><td>0.04</td></tr>
<tr><td>5</td><td>VP1:THR 102</td><td></td><td>39.35</td><td>30.65 ||||||||</td><td>0.23</td></tr>
<tr><td>6</td><td>VP1:MET 112</td><td></td><td>9.02</td><td>6.84 ||||||||</td><td>0.11</td></tr>
<tr><td>7</td><td>VP1:PHE 120</td><td></td><td>7.23</td><td>5.94 |||||||||</td><td>0.10</td></tr>
<tr><td>8</td><td>VP1:THR 122</td><td></td><td>13.88</td><td>13.88 ||||||||||</td><td>0.22</td></tr>
<tr><td>9</td><td>VP1:ILE 124</td><td></td><td>24.53</td><td>24.53 |||||||||||</td><td>0.39</td></tr>
<tr><td>10</td><td>VP1:ILE 126</td><td></td><td>2.93</td><td>2.93 |||||||||||</td><td>0.05</td></tr>
<tr><td>11</td><td>VP1:ALA 150</td><td></td><td>4.59</td><td>4.59 |||||||||||</td><td>0.02</td></tr>
<tr><td>12</td><td>VP1:TYR 152</td><td></td><td>31.49</td><td>11.93 ||||</td><td>0.16</td></tr>
<tr><td>13</td><td>VP1:PRO 174</td><td></td><td>78.80</td><td>10.04 ||</td><td>0.15</td></tr>
<tr><td>14</td><td>VP1:SER 175</td><td></td><td>42.12</td><td>4.61 ||</td><td>0.06</td></tr>
<tr><td>15</td><td>VP1:VAL 176</td><td></td><td>36.51</td><td>1.42 |</td><td>0.01</td></tr>
<tr><td>16</td><td>VP1:LEU 187</td><td></td><td>65.60</td><td>20.18 ||||</td><td>0.32</td></tr>
<tr><td>17</td><td>VP1:ILE 189</td><td></td><td>40.66</td><td>22.36 ||||||</td><td>0.36</td></tr>
<tr><td>18</td><td>VP1:MET 192</td><td></td><td>66.17</td><td>7.20 ||</td><td>0.29</td></tr>
<tr><td>19</td><td>VP1:TYR 198</td><td></td><td>29.52</td><td>26.61 ||||||||||</td><td>0.36</td></tr>
<tr><td>20</td><td>VP1:ALA 199</td><td></td><td>16.11</td><td>4.74 |||</td><td>-0.03</td></tr>
<tr><td>21</td><td>VP1:ASN 200</td><td></td><td>16.00</td><td>8.75 ||||||</td><td>-0.02</td></tr>
<tr><td>22</td><td>VP1:ASN 222</td><td></td><td>57.41</td><td>16.28 |||</td><td>-0.02</td></tr>
<tr><td>23</td><td>VP1:MET 224</td><td></td><td>32.51</td><td>20.07 |||||||</td><td>0.34</td></tr>
<tr><td>24</td><td>VP1:LEU 227</td><td></td><td>20.09</td><td>20.09 |||||||||||</td><td>0.32</td></tr>
<tr><td>25</td><td>VP1:VAL 246</td><td></td><td>3.02</td><td>3.02 ||||||||||</td><td>0.05</td></tr>
<tr><td>26</td><td>VP1:MET 248</td><td></td><td>11.45</td><td>11.45 |||||||||||</td><td>0.42</td></tr>
<tr><td>27</td><td>VP3:ALA 24</td><td></td><td>89.81</td><td>22.30 |||</td><td>0.36</td></tr>
<tr><td>28</td><td>VP3:PHE 25</td><td></td><td>144.72</td><td>12.67 |</td><td>0.20</td></tr>
<tr><td>29</td><td>VP3*:LEU 14</td><td></td><td>89.83</td><td>14.34 ||</td><td>0.23</td></tr>
</table>

B

Interfacing residues

Inaccessible residues
Solvent-accessible residues

HSDC Residues making **H**ydrogen/**D**isulphide bond, **S**alt bridge or **C**ovalent link
Interfacing residues

ASA Accessible Surface Area, Å² **BSA** Buried Surface Area, Å² **Δ^iG** Solvation energy effect, kcal/mol |||| Buried area percentage, one bar per 10%

<table>
<tr><th>##</th><th>Structure 1</th><th>HSDC</th><th>ASA</th><th>BSA</th><th>Δ^iG</th></tr>
<tr><td>VP1</td><td>Pleconaril:W11 400</td><td>H</td><td>618.96</td><td>577.39 ||||||||||</td><td>-2.08</td></tr>
<tr><td>VP3</td><td>Pleconaril:W11 400</td><td></td><td>618.96</td><td>41.57 |</td><td>0.05</td></tr>
<tr><td>VP3*</td><td>Pleconaril:W11 400</td><td></td><td>618.96</td><td>20.13 |</td><td>-0.40</td></tr>
</table>

<table>
<tr><th>##</th><th>Structure 2</th><th>HSDC</th><th>ASA</th><th>BSA</th><th>Δ^iG</th></tr>
<tr><td>1</td><td>VP1:VAL 74</td><td></td><td>4.22</td><td>3.01 ||||||||</td><td>0.05</td></tr>
<tr><td>2</td><td>VP1:TRP 98</td><td></td><td>19.11</td><td>9.07 |||||</td><td>0.15</td></tr>
<tr><td>3</td><td>VP1:LEU 100</td><td></td><td>38.64</td><td>32.58 |||||||||</td><td>0.52</td></tr>
<tr><td>4</td><td>VP1:ASN 101</td><td></td><td>34.16</td><td>7.80 |||</td><td>0.05</td></tr>
<tr><td>5</td><td>VP1:THR 102</td><td></td><td>40.48</td><td>31.61 ||||||||</td><td>0.28</td></tr>
<tr><td>6</td><td>VP1:MET 112</td><td></td><td>8.51</td><td>6.67 ||||||||</td><td>0.11</td></tr>
<tr><td>7</td><td>VP1:PHE 120</td><td></td><td>6.60</td><td>5.47 |||||||||</td><td>0.09</td></tr>
<tr><td>8</td><td>VP1:THR 122</td><td></td><td>13.38</td><td>13.38 ||||||||||</td><td>0.21</td></tr>
<tr><td>9</td><td>VP1:ILE 124</td><td></td><td>24.20</td><td>24.20 |||||||||||</td><td>0.39</td></tr>
<tr><td>10</td><td>VP1:ILE 126</td><td></td><td>3.01</td><td>3.01 |||||||||||</td><td>0.05</td></tr>
<tr><td>11</td><td>VP1:ALA 150</td><td>H</td><td>4.75</td><td>4.75 |||||||||||</td><td>0.01</td></tr>
<tr><td>12</td><td>VP1:TYR 152</td><td>H</td><td>30.39</td><td>14.59 |||||</td><td>0.21</td></tr>
<tr><td>13</td><td>VP1:PRO 174</td><td>H</td><td>75.48</td><td>11.13 ||</td><td>0.15</td></tr>
<tr><td>14</td><td>VP1:SER 175</td><td></td><td>41.57</td><td>4.89 ||</td><td>0.07</td></tr>
<tr><td>15</td><td>VP1:VAL 176</td><td>H</td><td>36.17</td><td>2.26 |</td><td>0.02</td></tr>
<tr><td>16</td><td>VP1:LEU 187</td><td></td><td>66.43</td><td>26.46 ||||</td><td>0.42</td></tr>
<tr><td>17</td><td>VP1:ILE 189</td><td></td><td>40.65</td><td>22.00 ||||||</td><td>0.35</td></tr>
<tr><td>18</td><td>VP1:MET 192</td><td></td><td>68.30</td><td>9.60 ||</td><td>0.38</td></tr>
<tr><td>19</td><td>VP1:TYR 198</td><td></td><td>28.61</td><td>25.16 |||||||||</td><td>0.33</td></tr>
<tr><td>20</td><td>VP1:ALA 199</td><td></td><td>16.01</td><td>3.50 |||</td><td>-0.03</td></tr>
<tr><td>21</td><td>VP1:ASN 200</td><td></td><td>15.39</td><td>1.11 |</td><td>-0.02</td></tr>
<tr><td>22</td><td>VP1:ASN 222</td><td></td><td>58.51</td><td>15.57 |||</td><td>-0.03</td></tr>
<tr><td>23</td><td>VP1:MET 224</td><td></td><td>32.57</td><td>20.91 |||||||</td><td>0.35</td></tr>
<tr><td>24</td><td>VP1:LEU 227</td><td></td><td>19.59</td><td>19.59 |||||||||||</td><td>0.31</td></tr>
<tr><td>25</td><td>VP1:VAL 246</td><td></td><td>2.85</td><td>2.85 ||||||||||</td><td>0.05</td></tr>
<tr><td>26</td><td>VP1:MET 248</td><td></td><td>12.93</td><td>12.60 ||||||||||</td><td>0.48</td></tr>
<tr><td>27</td><td>VP3:ALA 24</td><td></td><td>89.51</td><td>20.27 |||</td><td>0.32</td></tr>
<tr><td>28</td><td>VP3:PHE 25</td><td></td><td>144.63</td><td>24.46 ||</td><td>0.39</td></tr>
<tr><td>29</td><td>VP3*:LEU 14</td><td></td><td>92.94</td><td>15.79 ||</td><td>0.25</td></tr>
</table>

Fig. S13. Molecular interfaces of EV70 and WIN51711 (A) and pleconaril (B). Tables show hydrogen bonds and list of interfacing residues with buried surfaces participating in virus–inhibitor interaction. Letters in table column HSDC indicate residues making (H) – hydrogen bond; (S) – salt bridge; (D) – disulphide bond; (C) - covalent link. The analysis was performed using PDBePISA.

A

Pleconaril

VP3

EV70	A24	F25	L14*
EVD68	V	L	L
HRV14	A	L	L
HRV16	A	L	M
cons.	h	h	h

VP1

EV70	V74	W98	L100	N101	T102	M112	F120	T122	I124	I126	A150	Y152	P174
EVD68	V	W	I	N	T	L	F	A	I	I	A	Y	A
HRV14	V	W	I	N	L	L	F	S	Y	I	A	F	P
HRV16	I	W	I	N	L	F	F	S	I	M	Y	Y	A
cons	l	**W**	l	**N**	h	h	**F**	s	h	h	h	**a**	s

EV70	S175	V176	L187	I189	M192	Y198	A199	N200	N222	M224	L227	V246	M248
EVD68	S	V	M	I	M	Y	S	V	(D)	I	L	V	M
HRV14	S	V	F	V	V	Y	N	C	N	M	M	V	H
HRV16	S	V	F	L	L	Y	Y	M	N	M	L	I	H
cons.	**S**	**V**	h	l	h	**Y**	.	.	p	h	h	l	h

B

WIN51711

Conservation legend:
a - aromatic
h - hydrophobic
l - aliphatic
s - small
t -turnlike
. - non-conserved

VP3

EV70	A24	F25	L14*
EV71	I	L	L
HRV14	A	L	L
CVA9	A	L	L
Pol3	A	I	L
cons.	h	h	L

VP1

EV70	V74	W98	L100	N101	T102	M112	F120	T122	I124	I126	A150	Y152	P174
EV71	V	W	I	D	I	V	F	A	F	F	Y	F	P
HRV14	V	W	I	N	L	L	F	S	Y	I	A	Y	P
CVA9	V	W	I	N	T	L	F	M	V	F	I	Y	P
Pol3	V	W	I	T	Y	L	F	M	F	F	I	Y	P
cons.	**V**	**W**	l	p	h	h	**F**	.	h	h	h	**a**	**P**

EV70	S175	V176	L187	I189	M192	Y198	A199	N200	N222	M224	L227	V246	M248
EV71	S	V	V	V	M	Y	Q	W	N	M	F	I	M
HRV14	S	V	F	V	V	Y	N	C	N	M	M	V	H
CVA9	S	I	M	I	I	Y	S	N	L	L	I	V	F
Pol3	S	I	I	V	V	Y	S	H	D	F	L	I	M
cons.	**S**	l	h	l	h	**Y**	t	.	.	h	h	l	h

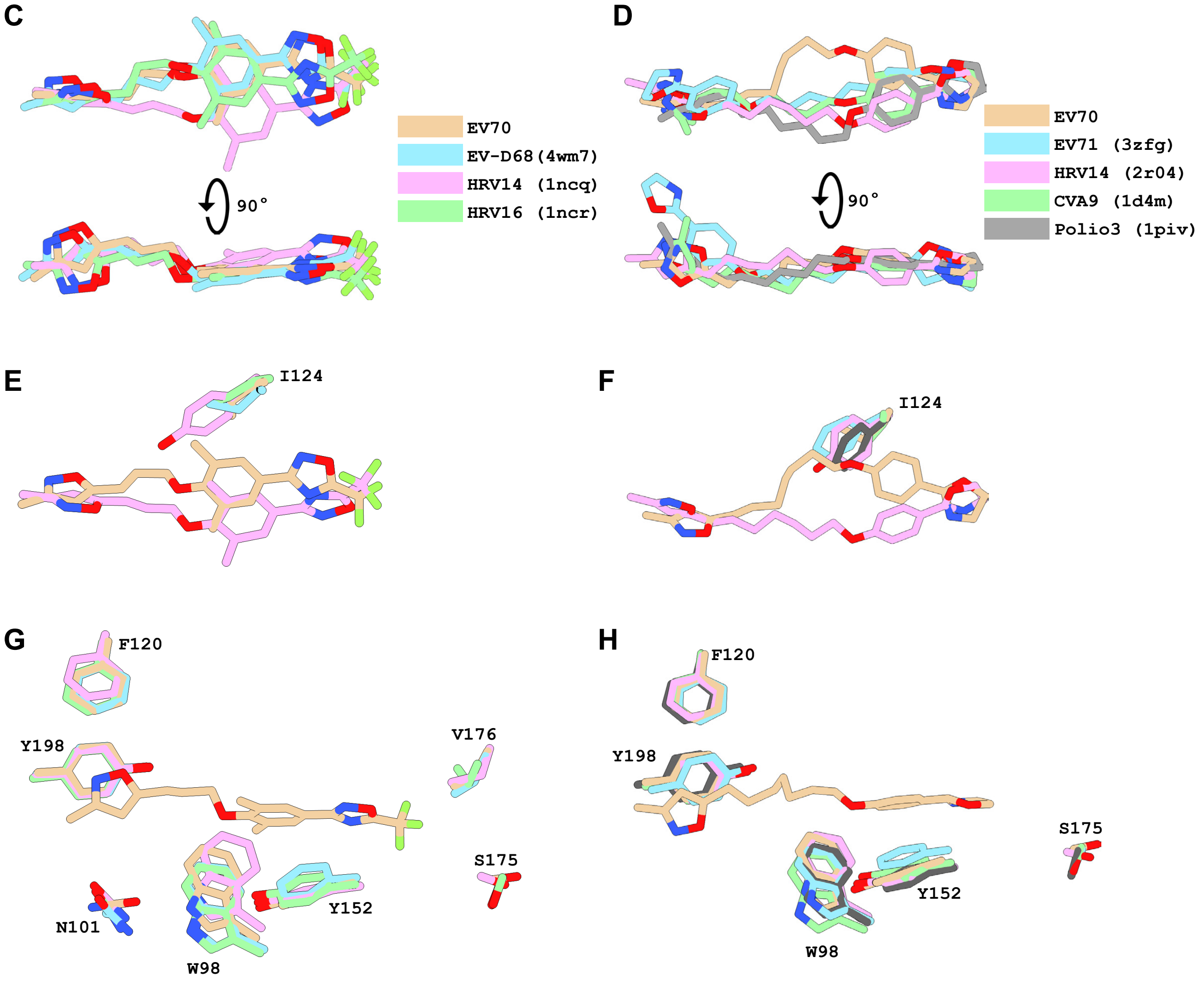

Fig. S14. Interaction of pleconaril and WIN51711 with other enteroviruses. VP1 structures of enteroviruses complexed with pleconaril or WIN51711 were structurally aligned to show the position of the inhibitor inside the hydrophobic pocket and to identify the homologous interacting amino acid residues in VP1. Tables show the conservation of interacting amino acids among known enterovirus structures in complex with pleconaril (A) and WIN51711 (B). The overall position of the pleconaril (C) and WIN51711 (D) inside the pocket is conserved, while the dislocation of the phenoxyl group is mainly mediated by clashes of aromatic residues in the homologous positions of EV70 Ile128. The position of absolutely conserved amino acid residues of already solved enterovirus- pleconaril (G) and WIN51711 (H) shows that the aromatic residues may contribute via stacking interactions to stabilize the virus-inhibitor complex. Enterovirus–inhibitor complexes were selected from up to date PDB records: EV-D68–pleconaril (pdb ID: 4wm7; (2)); HRV14–pleconaril (pdb ID: 1ncq; (6)); HRV16–pleconaril (pdb ID: 1ncr; (6)); EV71–WIN51711 (pdb ID: 3zfg; (7)); HRV14–WIN51711 (pdb ID:2r04; (8)); CVA9–WIN51711 (pdb ID: 1d4m; (9)); Polio3–WIN51711 (pdb ID: 1piv; (10))

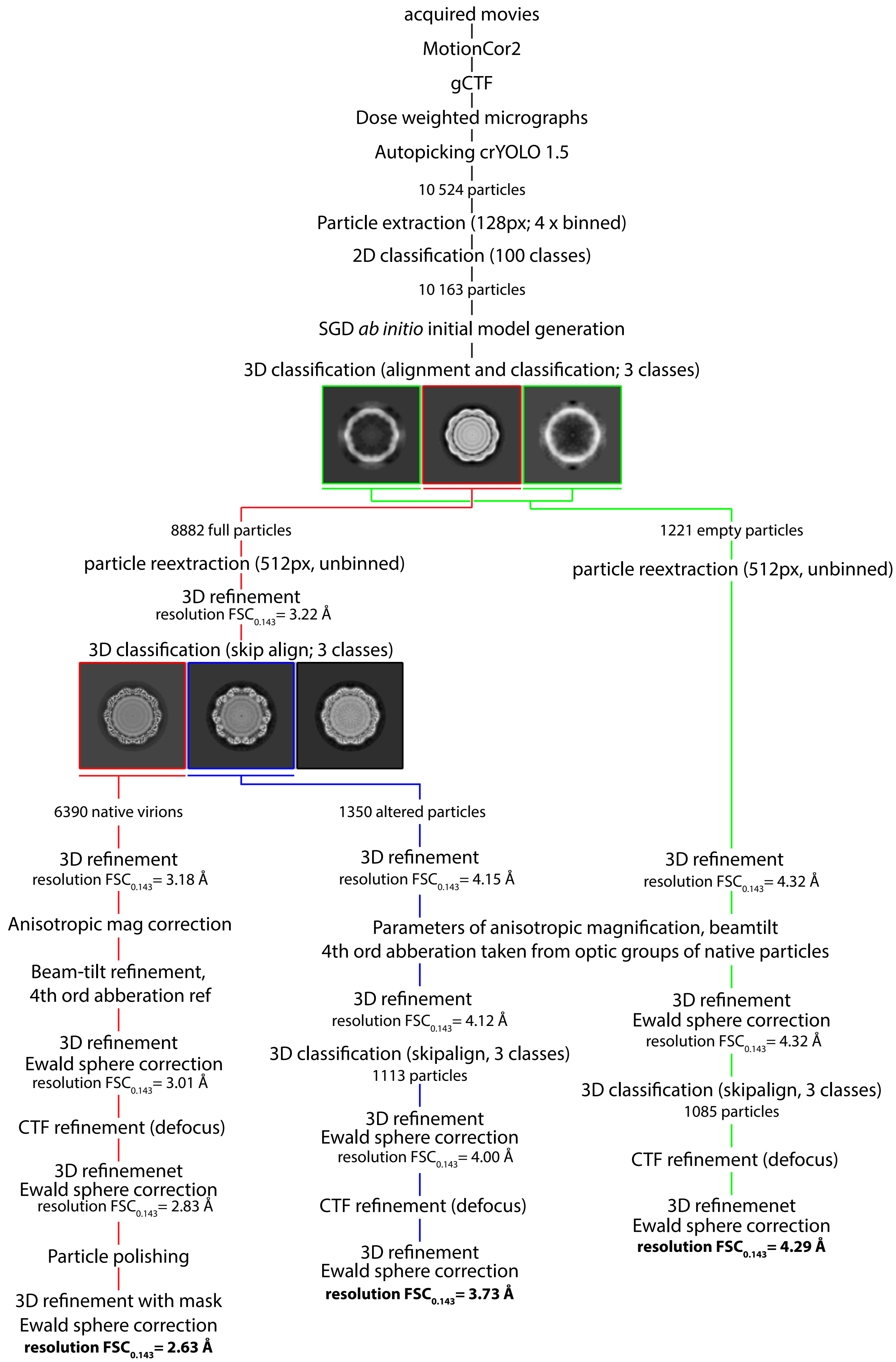

Fig. S15. Flowcharts of cryo-electron microscopic reconstruction workflow used for reconstruction of native, altered and empty EV70 particles.

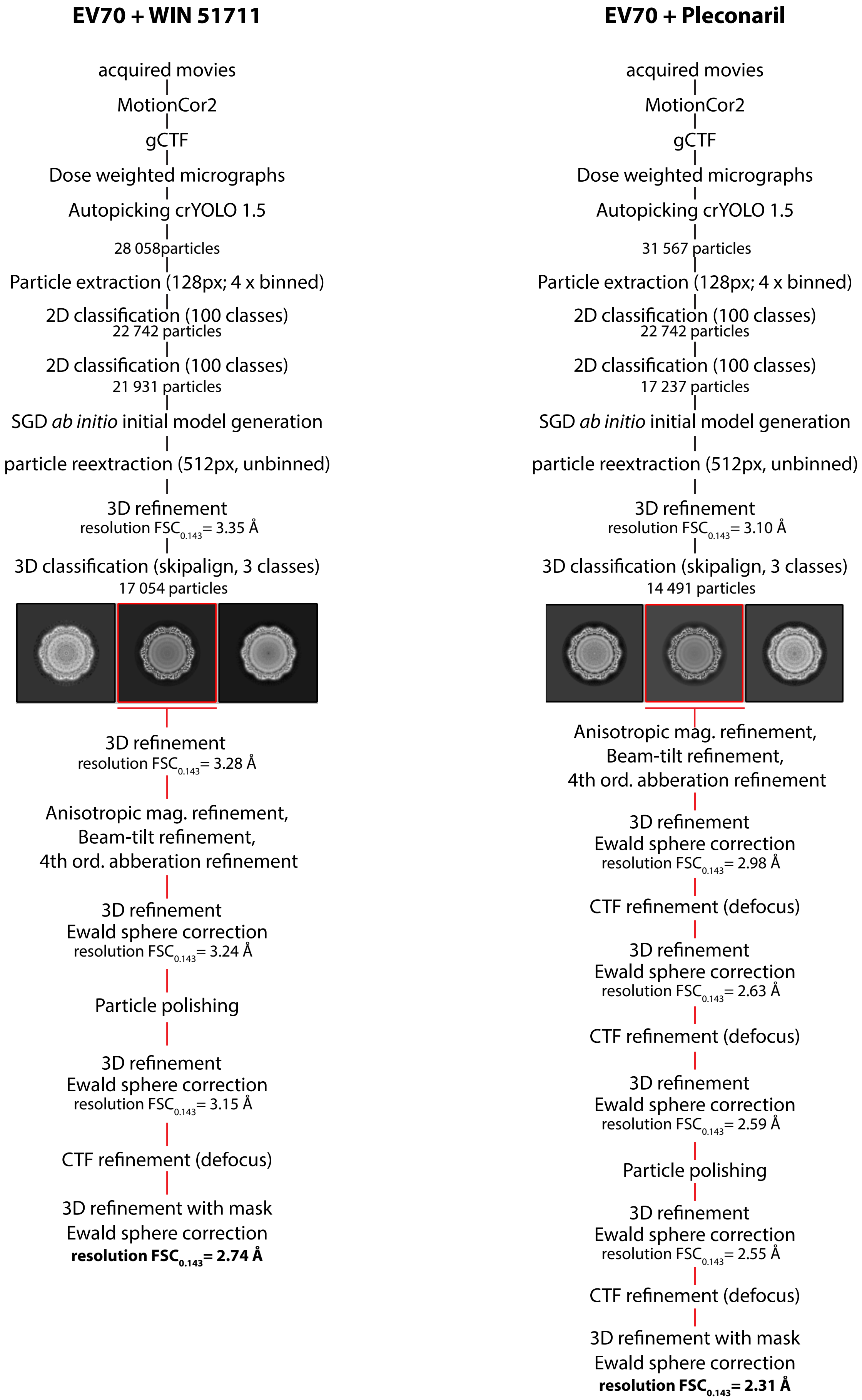

Fig. S16. Flowcharts of cryo-electron microscopic reconstruction workflow used for reconstruction of EV70 particles in complex with WIN51711 and pleconaril inhibitor.

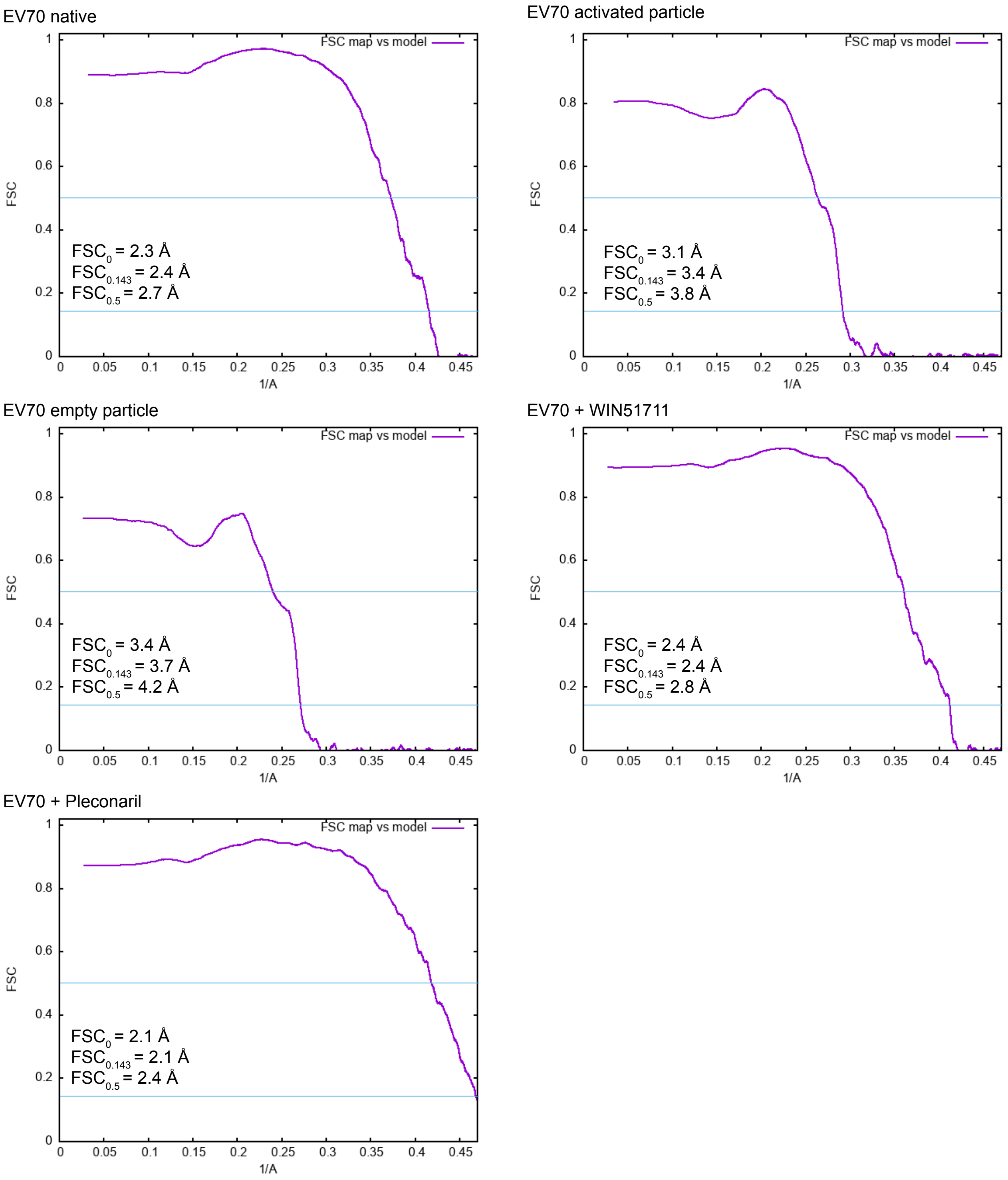

Fig. S17. Fourier shell correlation curves comparing final molecular structures to respective cryo-EM reconstructions. The resolution values at selected (0; 0.143; 0.5) FSC thresholds are shown in the plots.

Table S1. Cryo-EM data collection, refinement, and validation statistics.

**Data collection and processing**	**EV70 native**	**EV70 activated particle**	**EV70 empty particle**	**EV70+WIN51711**	**EV70+Pleconaril**
Detector	Falcon2	Falcon2	Falcon2	Falcon2	Falcon3
Detector acquisition mode	linear	linear	linear	linear	linear
Nominal Magnification	75,000	75,000	75,000	75,000	75,000
Voltage (kV)	300	300	300	300	300
Electron exposure on sample (e^-/Å^2)	48	48	48	48	46.8
Target defocus range (μm)	-3.0 – -1.0	-3.0 – -1.0	-3.0 – -1.0	-3.0 – -1.0	-3.0 – -1.0
Calibrated pixel size (Å)	1.063	1.063	1.063	1.061	1.061
Symmetry imposed	Icosahedral	Icosahedral	Icosahedral	Icosahedral	Icosahedral
Number of collected movies	2690;4008	2690;4008	2690;4008	2581	3594
Fractions per movie	7;16	7;16	7;16	16	39
Initial particle images	10 524	10 524	10 524	28 058	31 567
Final particle images	6390	1350	1221	17 054	14 491
Map resolution at FSC=0.143 (Å)	2.63	3.73	4.29	2.74	2.31
**Refinement and validation statistics**					
Map sharpening *B* factor (Å^2)	-48.9	-62.7	-105.2	-64.5	-52
Model composition					
Non-hydrogen atoms (one protomer)	6452	5195	4649	6455	6663
Protein residues (one protomer)	810	661	589	819	819
Waters (one protomer)	150	0	0	69	264
B factors					
Protein (Å^2)	43	76	110	42	41
Waters (Å^2)	56	N/A	N/A	53	58
R.M.S. deviations					
Bond lengths (Å)	0.011	0.012	0.012	0.005	0.014
Bond angles (°)	1.292	1.514	2.023	1.057	1.726
Validation					
Molprobity score*	1.84 (98th percentile)	1.75 (100th percentile)	2.47 (99th percentile)	1.88 (99th percentile)	1.49 (99th percentile)
Clashscore*	13.9 (93rd percentile)	6.79 (100st percentile)	15.42 (97th percentile)	9.94 (98th percentile)	3.24 (100th percentile)
EMringer score	4.58	2.71	2.12	3.54	5.0
Rotamers					
Favored (%)	99.6	97.7	91	96.9	96
Poor (%)	0	0.18	2.2	1.3	0.14
Ramachandran plot					
Favored (%)	96.9	94.5	90.9	95.9	94.6
Allowed (%)	3.1	5.5	8.9	4.1	5.3
Poor (%)	0	0	0.2	0	0.12
Accession codes					
PDB	7OPX	7OZI	7OZJ	7OZL	7OZK
EMDB	EMD-13022	EMD-13125	EMD-13126	EMD-13128	EMD-13127

* values according to Molprobity(11).

Table S2. Anti-enterovirus activity of WIN51711 and pleconaril.

Inhibitor	Virus	Cell line	EC_{50} [μM]	Reference
WIN51711	Poliovirus 1	HeLa	0.09	ref(12)
	Poliovirus 2	HeLa	0.01	ref(12)
	Poliovirus 3	HeLa	0.02	ref(12)
	CVA9	LLC-MK_2	0.5	ref(12)
	Echovirus 9 (K-16)	Vero	0.03	ref(12)
	Echovirus 9 (Barty)	Vero	0.06	ref(12)
	Echovirus 11	Vero	0.175	ref(12)
	Echovirus 12	Vero	0.09	ref(12)
	Enterovirus 70	Flow 2000	0.13	ref(12)
	**Enterovirus 70**	**hTERT RPE1**	**0.26**	**this study**
	Enterovirus 71	RD	600	ref(7)
	HRV2	-	3.5	ref(13)
	HRV3	HeLa	0.26	ref(12)
	HRV14	HeLa	0.175	ref(12)
	HRV14	-	0.4	ref(13)
	HRV16	HeLa	0.1	ref(12)
Pleconaril	Poliovirus 1	HeLa	>100	ref(14)
	Poliovirus 2	HeLa	1.1	ref(14)
	Poliovirus 3	HeLa	0.22	ref(14)
	Enterovirus 3	RD	0.31	ref(15)
	Enterovirus 4	RD	0.02	ref(15)
	Enterovirus 5	RD	1.05	ref(15)
	Enterovirus 6	RD	0.05	ref(15)
	Enterovirus 7	RD	0.05	ref(15)
	Enterovirus 9	RD	0.18	ref(15)
	Enterovirus 11	RD	0.01	ref(15)
	Enterovirus 24	RD	0.01	ref(15)
	Enterovirus 30	RD	0.01	ref(15)
	Enterovirus D68	HeLa	0.43	ref(2)
	**Enterovirus 70**	**hTERT RPE1**	**0.45**	**this study**
	Enterovirus 71	HeLa	>262	ref(16)
	CVA9	HeLa	0.005	ref(15)
	CVB1	LLC-MK_{2D}	0.002	ref(15)
	CVB2	LLC-MK_{2D}	0.003	ref(15)
	CVB3M	LLC-MK_{2D}	0.02	ref(15)
	CVB4	LLC-MK_{2D}	0.05	ref(15)
	CVB5	LLC-MK_{2D}	0.001	ref(15)
	HRV16	HeLa	0.57	ref(17)
	HRV14	HeLa	0.16	ref(17)

More detailed EC_{50} of WIN51711 on HRV strains available in ref(12)
More detailed EC_{50} of Pleconaril on various enterovirus clinical isolates available in ref(15)
More detailed table of EC_{50} of Pleconaril on EV-D68 clinical isolates in ref(18)

References

1. Landau M, et al. (2005) ConSurf 2005: The projection of evolutionary conservation scores of residues on protein structures. *Nucleic Acids Research* 33(SUPPL. 2):299–302.
2. Liu Y, et al. (2015) Structure and inhibition of EV-D68, a virus that causes respiratory illness in children. *Science* 347(6217):71–74.
3. Liu Y, et al. (2018) Molecular basis for the acid-initiated uncoating of human enterovirus D68. *Proceedings of the National Academy of Sciences* 115(52):E12209–E12217.
4. Sievers F, et al. (2011) Fast, scalable generation of high-quality protein multiple sequence alignments using Clustal Omega. *Molecular systems biology* 7(1):539.
5. Brown NP, Leroy C, Sander C (1998) MView: A web-compatible database search or multiple alignment viewer. *Bioinformatics* 14(4):380–381.
6. Zhang Y, et al. (2004) Structural and Virological Studies of the Stages of Virus Replication That Are Affected by Antirhinovirus Compounds. *Journal of Virology* 78(20):11061–11069.
7. Plevka P, et al. (2013) Structure of human enterovirus 71 in complex with a capsid-binding inhibitor. *Proceedings of the National Academy of Sciences* 110(14):5463–5467.
8. Badger J, Minor I, Oliveira MA, Smith TJ, Rossmann MG (1989) Structural analysis of antiviral agents that interact with the capsid of human rhinoviruses. *Proteins: Structure, Function, and Bioinformatics* 6(1):1–19.
9. Hendry E, et al. (1999) The crystal structure of coxsackievirus A9: new insights into the uncoating mechanisms of enteroviruses. *Structure* 7(12):1527–1538.
10. Hiremath CN, Grant RA, Filman DJ, Hogle JM (1995) Binding of the antiviral drug WIN51711 to the sabin strain of type 3 poliovirus: structural comparison with drug binding in rhinovirus 14. *Acta Crystallographica Section D Biological Crystallography* 51(4):473–489.
11. Chen VB, et al. (2010) MolProbity: All-atom structure validation for macromolecular crystallography. *Acta Crystallographica Section D: Biological Crystallography* 66(1):12–21.
12. Otto MJ, et al. (1985) In vitro activity of WIN 51711, a new broad-spectrum antipicornavirus drug. *Antimicrobial Agents and Chemotherapy* 27(6):883–886.
13. Smith TJ, et al. (1986) The site of attachment in human rhinovirus 14 for antiviral agents that inhibit uncoating. *Science* 233(4770):1286–1293.
14. De Palma AM, et al. (2008) Potential use of antiviral agents in polio eradication. *Emerging Infectious Diseases* 14(4):545–551.
15. Pevear DC, Tull TM, Seipel ME, Groarke JM (1999) Activity of Pleconaril against Enteroviruses. *Antimicrobial Agents and Chemotherapy* 43(9):2109–2115.
16. Tijsma A, et al. (2014) The capsid binder vapendavir and the novel protease inhibitor SG85 inhibit enterovirus 71 replication. *Antimicrobial Agents and Chemotherapy* 58(11):6990–6992.
17. Ledford RM, et al. (2004) VP1 Sequencing of All Human Rhinovirus Serotypes:Insights into Genus Phylogeny and Susceptibility to AntiviralCapsid-BindingCompounds. *Journal of Virology* 78(7):3663–3674.
18. Sun L, et al. (2015) Antiviral Activity of Broad-Spectrum and Enterovirus-Specific Inhibitors against Clinical Isolates of Enterovirus D68. *Antimicrobial Agents and Chemotherapy* 59(12):7782–7785.
